# The impact of financial burden on quality of life among German head and neck cancer survivors

**DOI:** 10.1186/s12885-025-13927-1

**Published:** 2025-03-20

**Authors:** Jonas Rast, Veit Zebralla, Theresa Wald, Andreas Dietz, Gunnar Wichmann, Susanne Wiegand

**Affiliations:** 1 Department of Otorhinolaryngology, Head and Neck Surgery, Christian-Albrechts-University of Kiel, 24105 Kiel, Germany; 2 Department of Otorhinolaryngology, Head and Neck Surgery, University Hospital Leipzig, Liebigstr. 10-14, 04103 Leipzig, Germany; 3 Compehensive Cancer Center Central Germany (CCCG), 04103 Leipzig, Germany

**Keywords:** Head and neck cancer, Financial burden, Financial toxicity, QoL, Impairments, Supportive care, Cancer survivorship

## Abstract

Head and neck cancer (HNC) survivors experience a significant financial burden (FB) after treatment, even in Germany with a statutory health insurance. The financial toxicity of cancer can cause higher morbidity and mortality rates but may also impair quality of life (QoL). Our investigation aims to elucidate the impact of HNC-related FB on QoL potentially facilitating better understanding of their interplay. Of *n* = 209 consecutive HNC patients attending our university hospital’s cancer aftercare program between August 2022 and March 2023, *n* = 200 patients completed the EORTC QLQ-Q30 questionnaire, and we analyzed the QoL scale data based on their self-reported FB. Parametric and non-parametric analyses were used to assess the impact of FB and independent predictors on QoL and QoL scales, and causal diagrams were used to visualize their causal relationship. HNC patients reported significant impaired QoL in consequence of FB. Significant detrimental effects of FB were observed on role functioning (RF; *p* = 0.0011), emotional functioning (EF; *p* = 0.0039), cognitive functioning (CF; *p* = 0.0149), and social functioning (SF; *p* = 0.0011). Advanced stage, advanced T category, and suffering from larynx/hypopharynx cancer demonstrated a significant quantitative interaction with FB increasing the risk for impaired QoL with respect to RF, EF, CF, and SF. HNC survivors suffer from significant impaired QoL and FB after treatment. In general, FB impairs particular QoL scales, and these QoL scales are differentially affected by particular tumor characteristics together with FB jointly impairing QoL of HNC survivors.

**Supplementary Information:**

The online version contains supplementary material available at 10.1186/s12885-025-13927-1.

## Background

Cancer patients have to overcome several barriers throughout their treatment and aftercare and are also significantly vulnerable to experience financial toxicity (FT) [1]. Although there have been remarkable advances in cancer therapies, these improvements are frequently accompanied by rising medical costs—particularly for radiation, chemotherapy, and emerging anti-neoplastic treatments [2–4]. However, these recent improvements in outcome of cancer patients are often linked to financial hardship, a phenomenon that persists even in countries with public healthcare systems and statutory health insurance. This problem has a multifactorial etiology: studies have identified factors such as out-of-pocket payments (OOPP), both in absolute terms and relative to income, as well as other challenges such as loss of income due to time off work, reduction in assets, the need to accept lower paid or alternative employment (or the inability to work at all), or maintaining the same workload [5–7]. Although many studies have been performed to assess the nature and impact of FT, a complete understanding of all the consequences and implications remains still elusive, particularly for patients with head and neck cancer (HNC). However, research investigating financial burden (FB) in HNC patients indicates significant associations between FB and diminished QoL, adverse disease prognosis, reduced contentment with oncological care, and compromised adherence to treatment protocols [8, 9]. Further, patients with HNC are linked to certain demographic features: They are underprivileged, more likely to be deprived, often less well-educated, usually have an impaired general health and nutritional status, and are less likely to return to work even after successful (curative) treatment than survivors of other cancers [7, 10, 11]. These associations consequently contribute to escalated healthcare expenses not only of those covered by the health insurance, thereby amplifying the susceptibility of HNC patients for FT [12, 13]. Although it is now known that German HNC patients face significant FB [10, 14, 15], the role of FB in relation to QoL in German patients with statutory health insurance is inadequately elucidated. Consequently, there is an urgent need for further research, particularly to investigate subgroups at increased risk for FB.

Indeed, we recently reported the FB due to OOPP or income loss experienced by patients of the same cohort as a result of their disease or its treatment [15]. The majority of the cohort (59.5%) experienced FB, with no observed differences in terms of age, sex, or type of insurance (statutory health insurance (SHI) *versus* private). The group that experienced FB had a higher proportion of individuals with OOPP compared to those experiencing income loss (50.0% *versus* 25.5%), as 68% of patients reporting loss in income also reported increased OOPP. The most common causes of OOPP were deductibles and transportation. Subgroups among HNC survivors with increased risk to experience FB are patients with locally advanced head and neck cancer (LAHNC), advanced Union for International Cancer Control (UICC) categories or those diagnosed with larynx/hypopharynx cancer. Both, OOPP and loss of income led patients to reduce spending on leisure, food and nutrition, housing and household expenses, as well as medical treatment and other services [15]. Our previous study [15] provided valuable insights into the FB of head and neck cancer patients. This study aims to investigate not only the direct economic impact, but also the subsequent impact on emotional, physical and social wellbeing to ultimately inform targeted interventions.

Previous studies, predominantly conducted in the United States of America, demonstrated the negative impact of FT in patients with cancer of various sites on QoL [2, 16]. Smith et al. showed that high levels of FT correlate with inferior QoL across most domains [17]. Therefore, cancer survivors suffer not only from objectively impaired organ function and organ-specific and general health resulting in an objective burden, but also from subjective distress with increased probability for psychological reactions and coping behaviors often deemed to be inadequate [18]. It is essential to emphasize that patients often consult their family members before making (treatment) decisions. This is particularly important as the economic burden of cancer affects the entire family [6]. Nevertheless, there is a lack of qualitative and quantitative studies exploring patients’ perspectives on financial coping mechanisms, though they would provide helpful information about how FB translates from higher costs to lower QoL and worse health outcomes [14]. Even more relevant could be knowledge about the interplay of FB and particular clinical characteristics of patients regarding their impact on QoL, as improved QoL is now accepted by the American and European authorities, US Food and Drug Administration (FDA) and European Medicines Agency (EMA), as an alternative primary outcome measure to overall survival for approval of particular treatments in randomized controlled trials (RCTs), and QoL is already established as sole primary endpoint in non-inferiority RCTs.

To assess the potential impact of FB on QoL among German HNC patients, we analyzed a large cohort from a cancer aftercare program at a university hospital in eastern Germany.

## Methods

### Statistical considerations and sample size

Information about the prospectively designed study including statistical considerations and sample size estimation required to investigate the impact of FB on QoL among HNSCC patients was recently published [15]. Briefly, by considering an error margin of 10% (*ε* = 0.1) and the 2-sided significance level of 5% (*u* = 1.96), and further an expected prevalence of toxic effects on income in a quarter of patients (i.e., *P* = 0.25), *n* = 73 cases would have been required at minimum to demonstrate a significant exposure of patients to FB. By calculating 25% dropouts caused by incomplete questionnaires, which should be compensated to prevent an underpowered study because of list-wise exclusion of cases, *n* = 98 patients each would be required for such a study with a binary distribution. Therefore, at least *n* = 196 patients should be enrolled.

### Study design and participants

The study was approved by the Ethics Committee of the Medical Faculty of the University Leipzig (vote 289722-ek) and was conducted in accordance with the ethical standards of the 1964 Declaration of Helsinki and its subsequent amendments. Enrolled were German-speaking adult patients who were treated at the Department of Otorhinolaryngology, Head and Neck Surgery and presented to the HNC aftercare program between August 2022 and March 2023. Patients participating in the aftercare program were invited to participate two to three months after completion of active HNC treatment. We consecutively invited patients aged 18 years and older during the weekly follow-up consultation until the predetermined number of informative cases (*n* > 196) was passed with *n* = 209 participants providing written informed consent. FB could be analyzed in *n* = 200 participants, as *n* = 9 accrued patients were excluded due to missing or inconsistent information on FB [15].

### Survey instrument

We conducted this study by handing out various questionnaires to patients attending their regular aftercare appointment. To capture various domains of QoL and behavioral consequences we used the EORTC QLQ-C30 [19], consisting of five functional scales (physical, role, cognitive, emotional, and social), along with three symptom scales (fatigue, pain, and nausea and vomiting), and a comprehensive global health and quality-of-life scale. The remaining individual items include prevalent symptoms frequently reported by cancer patients, such as dyspnea, appetite loss, sleep disturbance, constipation, and diarrhea, as well as financial difficulties. For assessment and interpretation of the results, we completely adhered to validated scales provided by Aaronson et al. [19].

Additionally, clinical and disease-related data were extracted from each patient’s records. The following patient parameters were evaluated: sex, age, and tumor site and stage, recurrence, and treatment regimens. To obtain information about the existence of FB in our cohort, the findings of a recent published analysis of the same cohort [15] were used, which is based on a self-administered questionnaire, containing questions on cancer-related out-of-pocket costs, monthly household income and income loss, employment status and consequences for social behavior (an English version of the questionnaire can be seen in the supplementary material (questionnaire 1)). All participating patients had sufficient knowledge of the German language in order to complete the questionnaire by themselves, with help provided by an assistant answering any arising questions on-site. The survey was completed only once by each patient.

### Statistical analysis

Distribution of data among groups were compared by chi-squared (*χ*^2^) tests for categorical data and homo- or heteroscedastic *t*-tests for parametric and the non-parametric *Mann-Whitney U*-or *Kruskal-Wallis* tests with *Bonferroni* correction for multiple testing, as appropriate. We analyzed *Likert*-type scales preferably using non-parametric tests, as the latter were shown to prevent over-fitting and leading to consistent results independent from data transformation [20], in particular *U* tests for sensitivity analysis of data obtained using parametric *t* tests and *Kruskal-Wallis* tests instead of ANOVA tests. Strength of effects was assessed using *Cohen’s d* according to convention. All analyses were done using SPSS version 29 (IBM Cooperation, Armonk, NY, USA), and 2-sided *p* values < 0.05 considered significant. We analyzed the impact of independent predictors (*Pi*) of FB, either LHSCC (laryngeal/hypopharyngeal squamous cell carcinoma *versus* other), advanced (*versus* early) stage, and T3 or T4 (*versus* T1 or T2) category on FB and each QoL domain using causal diagrams [21].

## Results

Adhering to the statistical considerations and the prospective design of accrual, 209 patients provided informed consent to participate in the study. The cohort comprised 200 patients with completed questionnaires eligible for statistical analysis. Table 1 summarizes the demographic information and characteristics of tumor localization, histology, tumor classification, and applied therapy modality for patients with and without FB.

**Table 1:** Cancer characteristics of the study participants. Numbers and percentages in groups with financial burden (FB) present or absent are shown with the particular odds ratio (OR) and 95% confidence interval (95% CI)

**Covariates**	Total, *n* = 200	FB present, *n* = 119	FB absent,*n* = 81	OR (95% CI)	*p* value^†^
	*n* (%)	*n* (%)	*n* (%)		
Sex					0.6325
Male	147 (73.5)	86 (43.0)	61 (30.5)	Ref. (0.629–1.590)	
Female	53 (26.5)	33 (16.5)	20 (10.0)	1.17 (0.614–2.231)	
Age Group					0.492772
18–50 years	14 (7.0)	9 (4.5)	5 (2.5)	Ref. (0.213–4.693)	
51–60 years	50 (25.0)	32 (16.0)	18 (9.0)	1.013 (0.294–3.486)	
61–70 years	81 (40.5)	50 (25.0)	31 (1 5.5)	1.116 (0.342–3.637)	
> 70 years	55 (27.5)	28 (14)	27 (13.5)	1.736 (0.515–5.846)	
Maritial status					0.4463
Single	27 (13.5)	17 (14.3)	10 (12.3)	Ref. (0.331–3.018)	
Married	121 (60.5)	70 (58.8)	51 (63.0)	0.807 (0.342–1.909)	
Widowed	16 (8.0)	8 (6.7)	8 (9.9)	0.588 (0.168–2.060)	
Living with partner	12 (6.0)	10 (8.4)	2 (2.5)	2.941 (0.533–16.22)	
Other	24 (12.0)	14 (11.8)	10 (12.3)	0.824 (0.267–2.540)	
Highest school degree					0.2542
General elementary education	77 (38.3)	46 (38.7)	31 (38.3)	Ref. (0.525–1.904)	
Intermediate vocational qualification or intermediate general qualification	61 (34.6)	33 (27.7)	28 (34.6)	0.794 (0.403–1.566)	
General maturity certificate	56 (22.2)	38 (31.9)	18 (22.2)	1.423 (0.691–2.930)	
Other	6 (3.0)	2 (1.7)	4 (4.9)	0.337 (0.058–1.954)	
Insurance status					0.4366
Private insured	8 (4.1)	6 (5.0)	2 (2.5)	Ref. (0.104–9.614)	
Statutory insured	186 (94.4)	110 (92.4)	76 (93.8)	0.482 (0.095–2.455)	
Other incl. free of charge coinsured	6 (3.0)	3 (2.5)	3 (3.7)	0.167 (0.009–2.984)	
**Treatment regimens**					
Surgery only					0.0558
No	150 (75.0)	95 (47.5)	55 (27.5)	Ref. (0.625–1.599)	
Yes	50 (25.0)	24 (12.0)	26 (13.0)	1.871 (0.980–3.572)	
Surgery + POR(C)T post-operative (adjuvant) radio-(chemo-)therapy					0.1367
No	91 (45.5)	49 (24.5)	42 (21.0)	Ref. (0.558–1.791)	
Yes	109 (54.5)	70 (35.0)	39 (19.5)	1.538 (0.871–2.716)	
Definitive radio- or radiochemotherapy					0.5963
No	167 (83.5)	98 (49.0)	69 (34.5)	Ref. (0.647–1.546)	
Yes	33 (16.5)	21 (10.5)	12 (6.0)	1.232 (0.569–2.670)	
N3 vs. other N category					0.3649
N0 - N2	180 (90.0)	72 (36.0)	108 (54.0)	Ref. (0.656–1.525)	
N3	7 (3.5)	3 (1.5)	4 (2.0)	2.000 (0.435–9.203)	
T category (7th ed.)					**0.012**
T1, T2 or T3	157 (78.5)	87 (43.5)	70 (35.0)	Ref. (0.641–1.561)	
T4	30 (15.0)	24 (12.0)	6 (3.0)	**3.218 (1.247–8.308)**	
T category (7th ed.)					**0.0008**
T1 or T2	128 (64.0)	65 (32.5)	63 (31.5)	Ref. (0.613–1.632)	
T3 or T4	72 (36.0)	54 (27.0)	18 (9.0)	**2.908 (1.539–5.493)**	
M category					0.7001
M0	183 (91.5)	109 (54.5)	74 (37.0)	Ref. (0.659–1.5 18)	
M1	4 (2.0)	2 (1.0)	2 (1.0)	1.473 (0.203–10.69)	
Tumor recurrence					0.0716
No	172 (86.0)	98 (49.0)	74 (37.0)	Ref. (0.653–1.532)	
Yes	28 (14.0)	21 (10.5)	7 (3.5)	2.265 (0.914–5.612)	
Larynx/hypopharynx vs. other localization					**0.0376**
Other localization	150 (75.0)	83 (41.5)	67 (33.5)	Ref. (0.634–1.577)	
Larynx/hypopharynx	50 (25.0)	36(18.0)	14 (7.0)	**2.076 (1.035–4.164)**	
Stage					**0.0186**
Early stage	56 (28.0)	26 (13.0)	30 (15.0)	Ref. (0.476–2.102)	
Advanced stage	131 (65.5)	85 (42.5)	46 (23.0)	**2.132 (1.129–4.027)**	
Stage by localization					**0.0009**
Early stage, other localization	36 (18.0)	17 (8.5)	19 (9.5)	Ref. (0.396–2.523)	
Advanced stage, other localization	103 (51.5)	60 (30.0)	43 (21.5)	1.560 (0.727–3.343)	
Early stage, larynx/hypopharynx	20 (10.0)	9 (4.5)	11 (5.5)	0.914 (0.305–2.740)	
Advanced stage, larynx/hypopharynx	28 (14.0)	25 (12.5)	3 (1.5)	**9.314 (2.379–36.458)**	

Table 1. reproduced by Rast et al., 2024, modified by Rast et al.

^**†**^*p* values from chi-squared (χ^2^) tests; Ref.: Reference category (mean OR = 1); POR(C)T: post-operative (adjuvant) radio(chemo)therapy; significant *p* values bold. Table reproduced from [15], modified

The EORTC QLQ-C30 questionnaire showed impaired QoL in German HNC patients. Patients who reported FB were at significantly higher risk of deteriorated QoL (OR = 5.144, 95%-CI 2.722–9.721; *p* < 0.0001). FB impairs each QLQ-C30 scale or domain (Table 2).

**Table 2:** Impact of financial burden on quality of life (QoL) measures according the EORTC QLQ-C30 questionnaire. Differences in mean values of QoL as well as functional scales and symptom scales were compared using *Cohen’s d* to assess the strength of impact of financial burden. *P* values shown are from 2-sided *Student’s t* tests for homo- or heteroscedastic distributions, as appropriate, and the non-parametric *Mann-Whitney U* test; *p* values < 0.05 tests are considered significant and bold

	Financial Burden	No Financial Burden						
	Mean (95% CI)	Mean (95% CI)	Cohen's d (95% CI)	Effect strength	*p* value^#^	*p* value^‡^		QoL
Global Health status / QoL								
Global Health status / QoL	58.94 (54.55–63.34)	65.42 (60.57–70.27)	0.285 (0.578 − 0.008)	small	0.057	0.0702	insignificant	impaired
**Functional scales**								
Physical functioning	71.09 (66.12–76.06)	77.22 (71.98–82.46)	0.243 (0.052–0.537)	small	0.1069	0.1199	insignificant	impaired
Role functioning	61.27 (55.00–67.53)	76.35 (70.03–82.67)	0.485 (0.184–0.784)	small	**0.0011**	**0.0026**	significant	impaired
Emotional functioning	71.78 (66.77–76.78)	81.89 (77.32–86.46)	0.416 (0.121–0.710)	small	**0.0039**	**0.0089**	significant	impaired
Cognitive functioning	79.55 (74.76–84.33)	87.45 (83.34–91.55)	0.345 (0.051–0.638)	small	**0.0149**	**0.0314**	significant	impaired
Social functioning	68.98 (62.79–75.17)	82.68 (77.47–87.89)	0.468 (0.172–0.764)	small	**0.0011**	**0.0068**	significant	impaired
**Symptom scales**								
Fatigue	38.86 (32.85–44.88)	25.07 (19.63–30.52)	0.475 (0.175–0.773)	small	**0.0011**	**0.0055**	significant	impaired
Nausea and vomitting	7.32 (3.64–11.01	0.68 (-0.10–1.43)	0.440 (0.139–0.739)	small	**0.0008**	**0.0019**	highly significant	impaired
Pain	35.34 (29.16–41.52)	24.57 (17.89–31.26)	0.336 (0.044–0.626)	small	**0.0239**	**0.0199**	significant	impaired
Dyspnoea	25.31 (19.24–31.38)	19.37 (13.34–25.40)	0.198 (0.099–0.494)	none	0.1754	0.3291	insignificant	impaired
Insomnia	38.89 (31.78–46.00)	22.37 (15.39–29.36)	0.472 (0.171–0.772)	small	**0.0014**	**0.0036**	significant	impaired
Appetite loss	24.45 (18.30–30.61)	9.13 (4.32–13.94)	0.540 (0.236–0.842)	moderate	**0.0002**	**0.0003**	highly significant	impaired
Constipation	7.16 (3.58–10.74)	4.57 (1.63–7.50)	0.156 (0.143–0.453)	none	0.2728	0.5538	insignificant	impaired
Diarrhoea	6.98 (3.19–10.75)	8.77 (3.67–13.87)	0.085 (0.377 − 0.208)	none	0.5707	0.6476	insignificant	not impaired
Financial difficulties	27.99 (22.10–33.87)	6.76 (3.21–10.31)	0.824 (0.514–1.132)	strong	**9.30 • 10** ^**− 9**^	**5.42 • 10** ^**− 7**^	highly significant	Impaired

*p* value^#^*p* value from parametric homo- or heteroscedastic *Student's t* test

*p* value^**‡**^*p* value from non-parametric rank sum (*Mann-Whitney U*) test

Upon analysis of the functional scales, a significant effect of FB was observed on role functioning (*p* = 0.00109), emotional functioning (*p* = 0.00389), cognitive functioning (*p* = 0.01495), and social functioning (*p* = 0.00109). However, no significant effect of FB was observed on physical functioning (*p* > 0.1).

When analyzing the symptom scales, there was no correlation between FB and dyspnea, constipation or diarrhea (all *p* > 0.1) but significant associations between FB and nausea and vomiting (*p* = 0.00075) and loss of appetite (*p* = 0.00017). Patients with HNC reporting FB also had an impaired QoL in terms of fatigue (*p* = 0.00106) and insomnia (*p* = 0.00140). Each category implied FB-associated impaired QoL. Each significant variable demonstrated a significant impact on the QoL of HNC patients, as revealed by both the Student’s *t*-test and the non-parametric rank sum (Mann-Whitney *U*) test (Table 2). These congruent statistical findings highlight the crucial role of FB in affecting the QoL of HNC patients. Additionally, highly significant predictors of FB [15], especially T category, UICC stage, and larynx/hypopharynx cancer, were found to have a modifying effect of FB on QoL (Table 3).

**Table 3:** Overview about heterogeneity in impact of independent predictors (Pi) of financial burden (FB) on quality of life (QoL) according to EORTC QLQ-C30 scales. Shown are Bonferroni-corrected *p*-values from Kruskal-Wallis tests for orthogonal comparison of QLQ-C30 scales in HNC patients categorized according to binary dichotomized covariates localization (larynx/hypopharynx versus other), stage (UICC I or II versus UICC III or IV), and T category (T1/T2 versus T3/T4) and FB (absent versus present). Significant *P* values are bold

	FB x Localization			FB x UICC stage			FB x T category		
Global Health status / QoL	*p* value‡	heterogeneity	QoL	*p* value‡	heterogeneity	QoL	*p* value‡	heterogeneity	QoL
Global Health status / QoL	0.2795	insignificant	not impaired	0.2379	insignificant	not impaired	0.0901	insignificant	not impaired
**Functional scales**									
Physical functioning	0.2290	insignificant	not impaired	0.1358	insignificant	not impaired	**0.0120**	significant	impaired
Role functioning	**0.0053**	significant	impaired	**0.0150**	significant	impaired	**0.0011**	significant	impaired
Emotional functioning	**0.0423**	significant	impaired	**0.0106**	significant	impaired	**0.0201**	significant	impaired
Cognitive functioning	0.1085	insignificant	not impaired	0.1380	insignificant	not impaired	0.1818	insignificant	not impaired
Social functioning	**0.0352**	significant	impaired	**0.0060**	significant	impaired	**0.0004**	significant	impaired
**Symptom scales**									
Fatigue	**0.0276**	significant	impaired	**0.0418**	significant	impaired	**0.0346**	significant	impaired
Nausea and vomitting	**0.0025**	highly significant	impaired	**0.0135**	highly significant	impaired	**0.0152**	highly significant	impaired
Pain	0.1395	insignificant	not impaired	**0.0429**	significa nt	impaired	0.0501	insignificant	not impaired
Dyspnoea	0.4740	insignificant	not impaired	0.5659	insignificant	not impaired	0.5234	insignificant	not impaired
Insomnia	**0.0298**	significant	impaired	**0.0475**	significant	impaired	**0.0242**	significant	impaired
Appetite loss	**0.0031**	highly significant	impaired	**0.0007**	highly significant	impaired	**0.0001**	highly significant	impaired
Constipation	0.6392	insignificant	not impaired	0.7385	insignificant	not impaired	0.3975	insignificant	not impaired
Diarrhoea	0.4385	insignificant	not impaired	0.7678	insignificant	not impaired	0.4495	insignificant	not impaired
Financial difficulties	**1.1·10 − 5**	highly significant	impaired	**4.0·10 − 6**	highly significant	impaired	**3.1·10 − 7**	highly significant	impaired

The non-parametric *Kruskal-Wallis* test revealed significant heterogeneity in various QoL scales according to orthogonal comparison of patients binary dichotomized into present or absent FB and 3 independent predictors (*Pi*) of FB, localization (LHSCC *versus* other), stage (advanced, UICC III or IV, *versus* early, I or II), and T category (T3 or T4 *versus* T1 or T2) (Table 3). Boxplots highlighting the different interaction of FB and the 3 *Pi* in the QoL scales and domains are shown in the supplementary Figures S1-S3, and Table S1 showing the respective *p* values for corresponding differences.

**Fig. 1:**
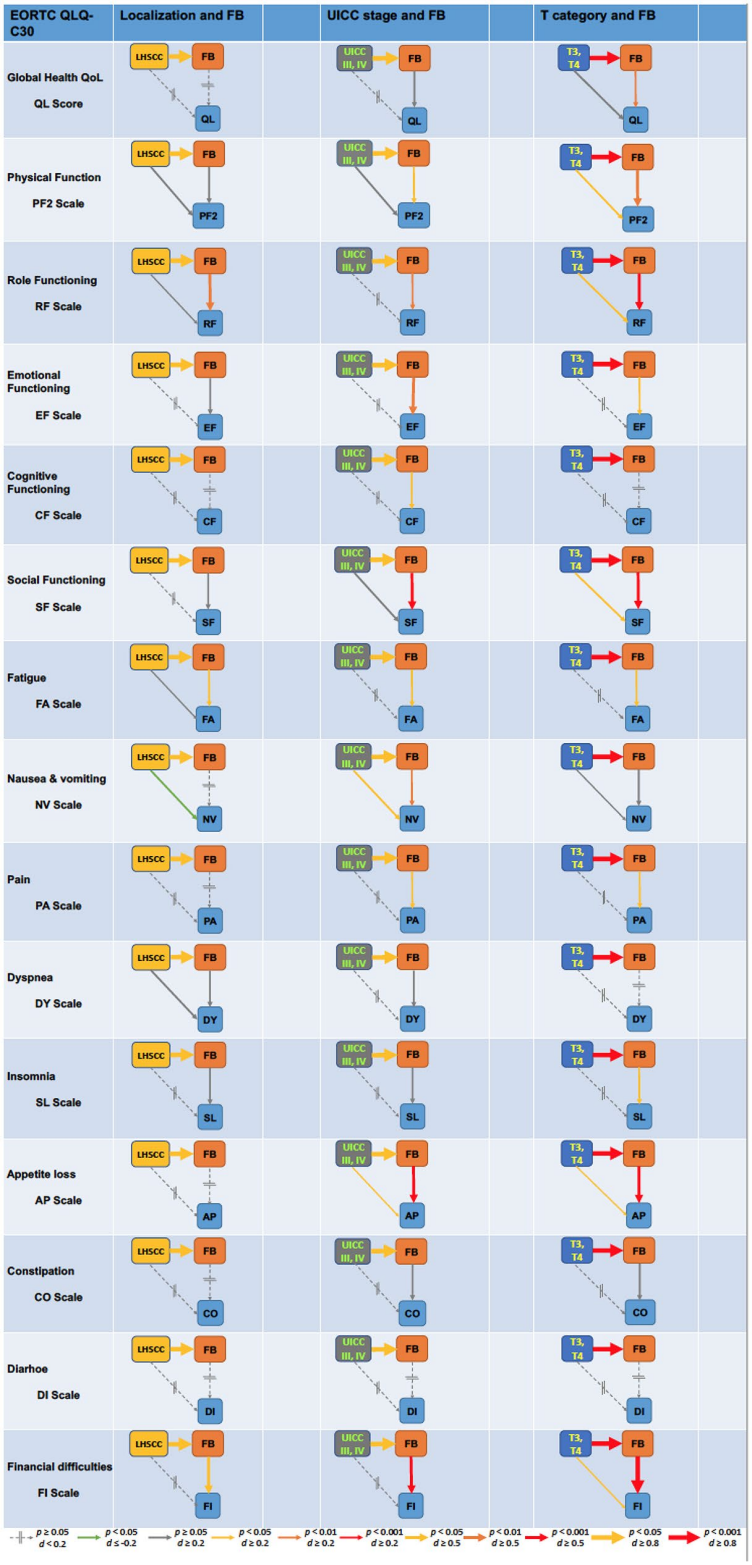
Causal diagrams demonstrating the strength in impact according to Cohen’s d of the independent predictors (Pi) of financial burden (FB), localization (left), stage (middle), and T category (right) on FB, and of FB on the 15 quality of life (QoL) measures of the EORTC QLQ-C30 questionnaires. Strength of effects (visualized through the size of arrows) and significance levels (color-coded according to the legend provided at the bottom) demonstrate the impact of the causal path that FB affects QoL exceeding the effect of the 3 independent predictors (localization, UICC stage and T category) that rather represent confounders regarding their impact on FB

To gain further information about the impact of FB on various QoL dimensions, we used causal diagrams. Figure 1 shows the impact of either LHSCC (*versus* other), advanced (*versus* early) stage, and T3 or T4 (*versus* T1 or T2) category on FB and each QoL domain. The causal diagrams in detail demonstrate the strong impact of FB on particular QoL scales either identical in strength or exceeding strength of effects exerted by the individual *Pi* alone accompanied by even lower *p* values (Fig. 1).

According to Shahar & Shahar [21], this indicates that the 3 independent predictors predicting FB are rather confounders regarding impaired QoL triggered by FB, as FB (pre-dominantly) affects most QoL domains with only dyspnea (DY Scale), constipation (CO Scale), and diarrhea (DI Scale) being the only exceptions. For instance, LHSCC and advanced stage are not responsible for financial difficulties according to FI scale, and only a small but significant effect on FI is associated with T3 or T4 categories. The significant association between LHSCC, advanced stage, and T3/T4 category and FB represent strong effects. The FB associated with LHSCC or advanced stage individually lead to moderate effects on FI, whereas the strong effect of T3/T4 categories on FB results in financial difficulties according to FI exceeding the threshold for strong effects (*Cohen’s d* ≥ 0.8).

To analyze whether either income loss or higher OOPP are responsible for poorer QoL, we used *t-tests* to gain information about effect sizes and significance. Overall, we found more moderate effects (*Cohen’s d* ≥ 0.5) and fewer null effects with overall higher significance for OOPP, as FI was the only QoL scale where loss of income demonstrated greater effect size (*Cohen’s d* = 1.037, 95%-CI 0.682–1.390, *p* = 6.87 · 10^− 6^*versus* 0.589, 95%-CI 0.289–0.887, *p* = 7.12 · 10^− 5^).

## Discussion

QoL of HNC patients as assessed using the EORTC QLQ-C30 was found to be strongly impaired through FB, and the independent predictors of FB, especially localization of the primary lesion in larynx or hypopharynx, advanced stage and T3 or T4 category, exert their detrimental impact on QoL through FB. In other words, FB affects QoL, representing the causal path, while LHSCC, advanced stage, and T3/T4 act as cofounders affecting FB and QoL. Causal path and confounding paths together contribute to the marginal (crude) association between FB, localization, stage and T category impairing QoL [21].

QoL refers to a patient’s subjective evaluation of how their disease impacts their daily activities. In individuals with advanced cancer, QoL is influenced by various medical and sociodemographic factors, in addition to the disease itself and its treatment [22, 23]. In a previous study we demonstrated strongly impaired QoL in HNC patients compared to the general population (QoL mean 53.8, 95%-CI 52.3–55.3 *versus* 73.0, 72.2–73.8; *p* < 0.0001) [24]. So far it was not clear, if FB is present in HNC patients in countries with publicly funded healthcare systems such as in many European countries, and particularly not how FB affects their QoL. We recently [15] reported FB in about 59.5% of patients linked to impaired QoL (OR = 5.14, 95%-CI 2.72–9.72; *p* < 0.0001) [15], and FB may have contributed to impaired QoL. However, despite reporting this outcome, at that time we neither understood well, how FB plays its impairing role regarding QoL, and even more not, how particular characteristics of HNC patients in presence of FB exert their impact on QoL in general or on individual QoL scales.

By using the well-established EORTC QLQ-C30 we found mean QoL 61.4 (95%-CI 58.2–64.6) and confirmed our earlier findings of significantly impaired QoL in HNC patients compared to the general population (73.0, 95%-CI 72.2–73.8; *p* < 0.0001) [24] but demonstrated also that patients experiencing FB enormously suffer from impaired QoL compared to HNC patients without FB (Table 2). These findings are in line with previous conducted research [25–28]. For example, Rogers et al. showed that FB was associated with worse physical and social-emotional functioning [29]. We extend these findings by reporting associations with small effects between cancer-related FB and impaired global health status, role and cognitive functioning, measured by the EORTC QLQ-C30. These findings support the increasing evidence that cancer-related FB and financial difficulties (FI) are linked to lowered QoL in various countries and healthcare environments [26]. However, the prevalence and extent of FB depends on the preexisting healthcare system and the varying insurance coverages of costs otherwise charged to the patient. Objective causes of financial hardship include cancer-related income loss and non-reimbursed OOPP. While most medical costs in Germany are covered by a person’s health insurance, it is now known that German cancer patients experience significant income loss and OOPP. We reported that HNC patients stating FB due to out-of-pocket payments had an average cost of 1.716 € per year related to their disease [15]. In contrast, a survey of 73 advanced HNC patients in Chicago reported even higher out-of-pocket costs of monthly $806 [30]. These differences in FB are not surprising, given the structural differences in both countries and their healthcare systems. Therefore, it is worth noting that in the US, the type of insurance has a decisive influence on the FB.

However, we showed that cancer related non-reimbursed OOPP are probably contributing more to diminished QoL of HNC patients experiencing FB than loss in income. Covering the additional cancer-related costs by compensating for OOPP might be able to improve patients’ QoL. Considering the common causes (deductibles, transportation) of OOPP [15], we expect an improved QoL of HNC patients through compensation of OOPP as reduced QoL among HNC patients probably results from uncompensated OOPP. Compared to other rather costly new interventions aiming on improving outcome, OS, QoL or both, a compensation for increasingly high OOPP might be cost-effective, especially if OOPP exceeding particular limits are covered focusing especially on the most vulnerable group among HNC patients with lowest income.

The finding that FB is a significant predictor of QoL highlights the potentially crucial impact of financial strain on patients’ overall well-being and cannot be ignored as the FB of cancer treatment themselves can significantly affect disease outcomes [8, 31], emphasizing once again the self-interest of insurance companies to provide financial compensation to their patients. Our findings could be highly relevant for future budget debates with insurance companies, policy makers and politicians involved in the health care system. In the previous study we showed that increased OOPP and loss of income have led patients to cut back on essential expenditures. Specifically, many patients reduced spending on leisure, food and nutrition, housing and household expenses, and even on medical treatments and other services. This suggests that the financial strain not only compromises patients’ quality of life but may also have deleterious effects on their overall health. For instance, cutting expenses on nutrition and housing could exacerbate malnutrition and health problems, while reducing spending on medical care might lead to poorer treatment adherence and outcomes.

Further, it is worth mentioning that our analysis revealed that the CF scale (cognitive functioning) showed a significant small effect in the main analysis with *p* > 0.1 in the three sub analyses. Neither stage, localization, nor T category in the presented cohort demonstrate a significant difference in the dependence of QoL regarding cognitive functioning from FB. Depending on FB, even patients with early stage tumors and lower T category, or outside larynx/hypopharynx reported reduced cognitive functioning. Additionally, the QLQ-C30 symptom scales also showed associations between cancer-related FB and impaired QoL regarding fatigue, nausea and vomiting, pain, insomnia, appetite loss, and financial difficulties. Whereas fatigue is a common symptom among cancer patients, it is hardly comprehensible and is not easy to treat [24]. ‘Appetite loss’ showed a highly significant moderate effect and may indicate the prevalent issue of malnutrition among HNC patients. The available data suggest a prevalence range of 30–50%, especially for HNC located in the oropharynx and the hypopharynx [32]. Malnutrition is a complex condition that can have multiple causes, including dysphagia, odynophagia, and reduced appetite induced by the tumor. Additionally, radio(chemo)therapy can exacerbate malnutrition due to mucositis, taste alteration, dysphagia, xerostomia, nausea and vomiting [32, 33]. However, our findings indicate that FB must also be taken into consideration when examining insufficient food intake. Malnutrition correlates with heightened chemoradiotherapy (CRT)-related toxicity and susceptibility to infections, resulting in prolonged treatment-package time due to interruptions, poorer clinical outcomes, heightened morbidity and mortality rates, and diminished QoL [32, 34, 35].

With some exceptions (compare Tables 2 and 3) we consistently found significant impaired QoL dependent on FB and hypothesized that FB by exerting an even stronger impact on QoL than its independent predictors T category, localization and UICC stage may indicate the causative path from FB to impaired QoL, and T3/T4, advanced stage and LHSCC being confounders in this regard.

Based on a large representative cohort of HNC patients we consistently were able to demonstrate a significant link between FB and impaired QoL, no matter if parametric homo- or heteroscedastic tests as well as non-parametric tests were used, and sensitivity analyses revealed stability of the link between FB and QoL with localization, tumor category and stage being relevant confounders. Utilizing causal diagrams, we were able to provide mechanistic insights by demonstrating that FB affecting QoL represents the causal path and the impact of LHSCC, advanced stage and T3/T4 on FB and QoL rather being confounding paths. The study included a diverse group of German HNC patients representing a broad socioeconomic cross-section. Therefore, the conclusions drawn from this study can be applied to German HNC patients broadly.

However, it is possible that there are additional potential confounders that were not analyzed in this study. Baili et al. suggest that distance to hospital, which is influenced by living situation (rural vs. urban), may influence the level of FB [36] and therefore we believe it may also influence QoL. Consequently, further research could clarify the complex interactions between FB and QoL.

## Conclusion

In this study of HNC patients, FB was associated with worse QoL across several domains. Thus, our findings highlight a gap in the care and support of HNC patients. The findings can inform cancer centers and treating physicians about the importance of screening for FB due to its potential consequences for the patient’s outcome throughout the treatment and hence may help to develop tailored tools and implementations to better address the potential needs of these underserved patients. There seems to be potential for improving QoL through financial counseling for cancer patients. Healthcare providers should refer HNC patients to appropriate support providers, such as social workers, psycho-oncologists or patient navigators, who can assist them with obtaining financial assistance improving QoL.

## Electronic supplementary material

Below is the link to the electronic supplementary material.

Supplementary Material 1

Supplementary Material 2

Supplementary Material 3

Supplementary Material 4

Supplementary Material 5

## Data Availability

The datasets used and/or analysed during the current study are available from the corresponding author on reasonable request.
