# Supplementary material for: The impact of financial burden on quality of life among German head and neck cancer survivors
Source: BMC Cancer. 2025 Mar 20;25:514. doi: 10.1186/s12885-025-13927-1 (PMC11927114; doi:10.1186/s12885-025-13927-1)
Supplement: Supplementary file 1 — Supplementary Material 1 [file 12885_2025_13927_MOESM1_ESM.pdf]

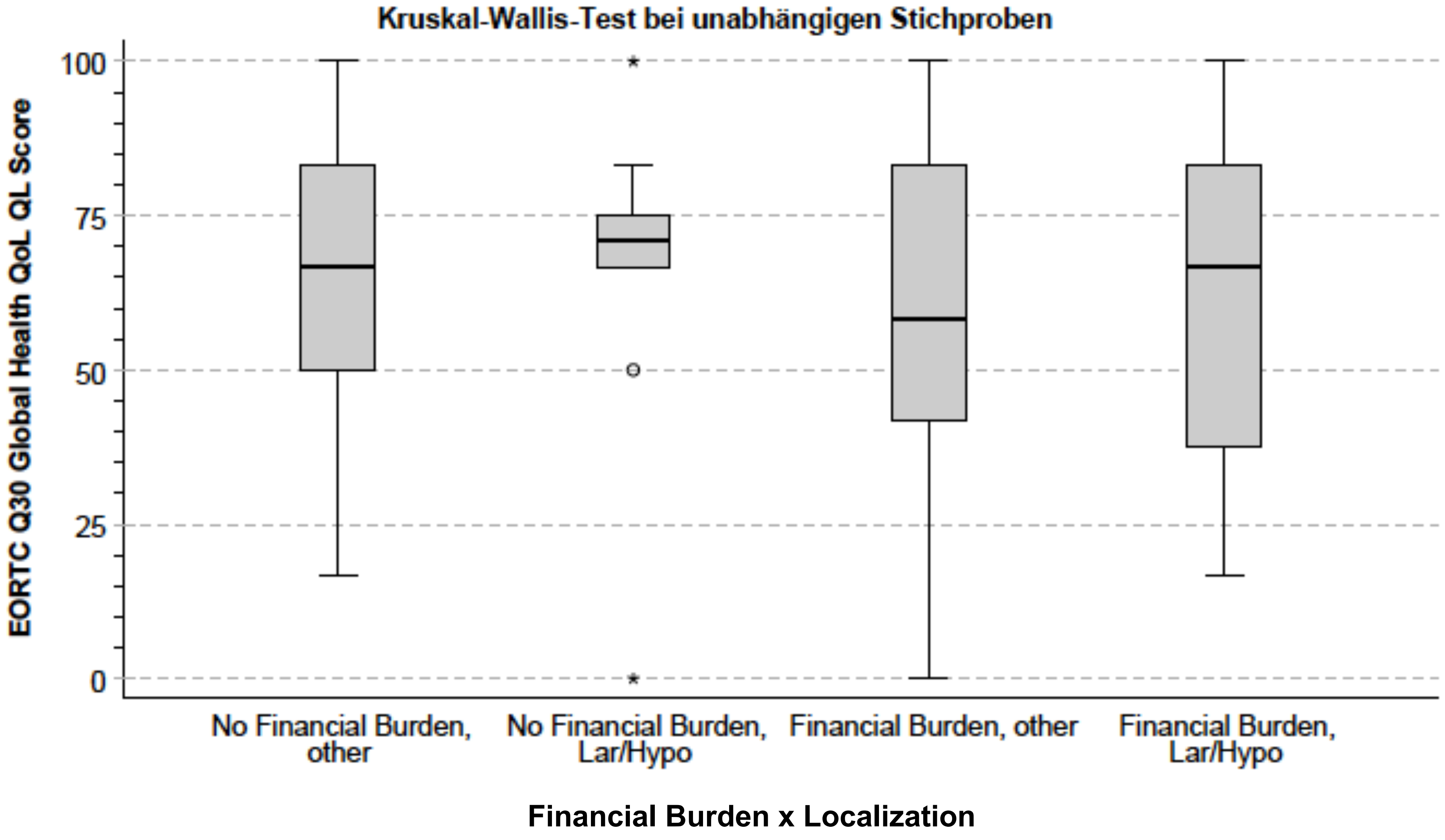
Kruskal-Wallis-Test bei unabhängigen Stichproben
EORTC Q30 Global Health QoL QL Score
100
75
50
25
0
No Financial Burden, other
No Financial Burden, Lar/Hypo
Financial Burden, other
Financial Burden, Lar/Hypo
Financial Burden x Localization

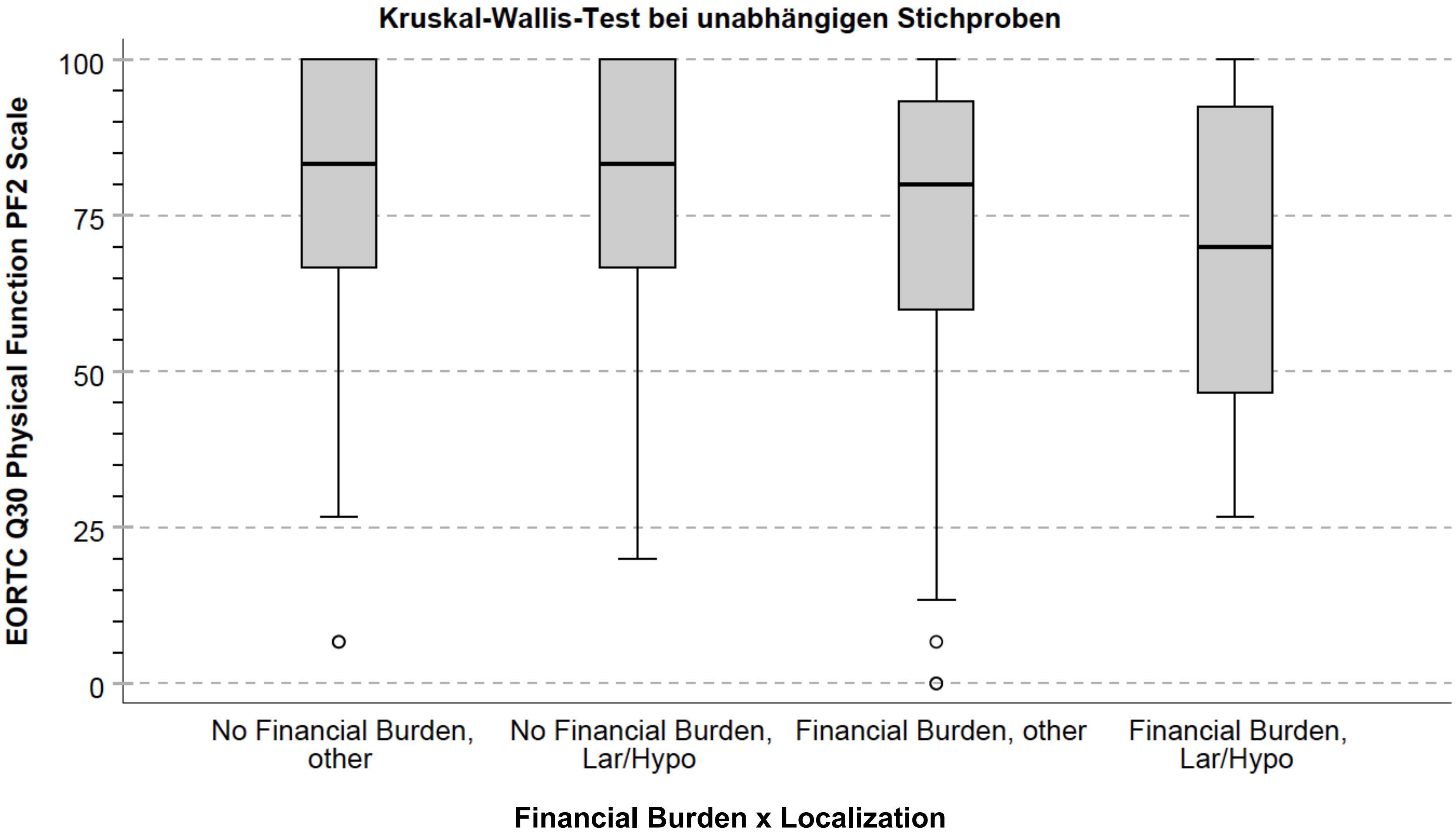
Kruskal-Wallis-Test bei unabhängigen Stichproben
EORTC Q30 Physical Function PF2 Scale
100
75
50
25
0
No Financial Burden, other
No Financial Burden, Lar/Hypo
Financial Burden, other
Financial Burden, Lar/Hypo
Financial Burden x Localization

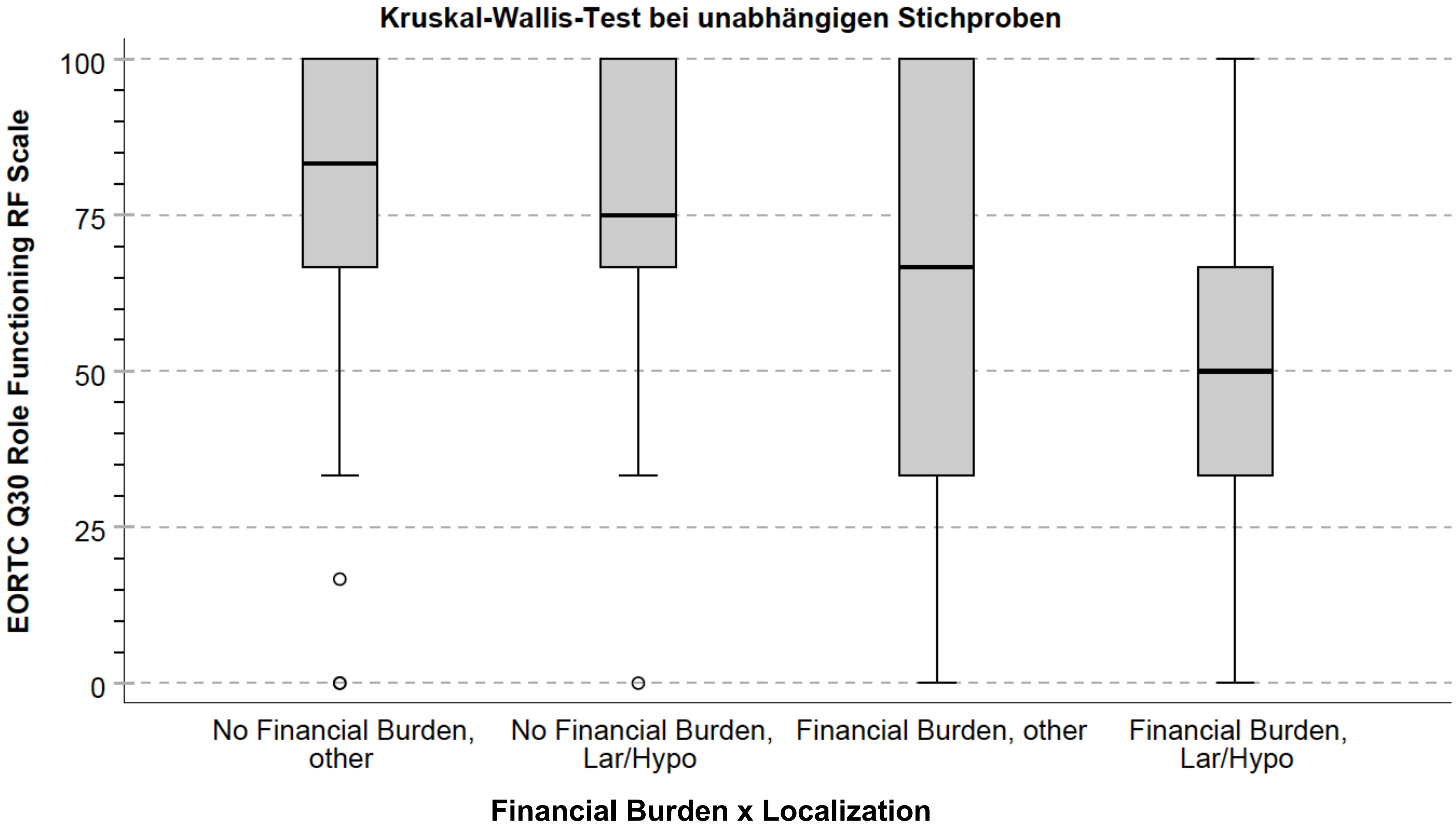
Kruskal-Wallis-Test bei unabhängigen Stichproben
EORTC Q30 Role Functioning RF Scale
100
75
50
25
0
No Financial Burden, other
No Financial Burden, Lar/Hypo
Financial Burden, other
Financial Burden, Lar/Hypo
Financial Burden x Localization

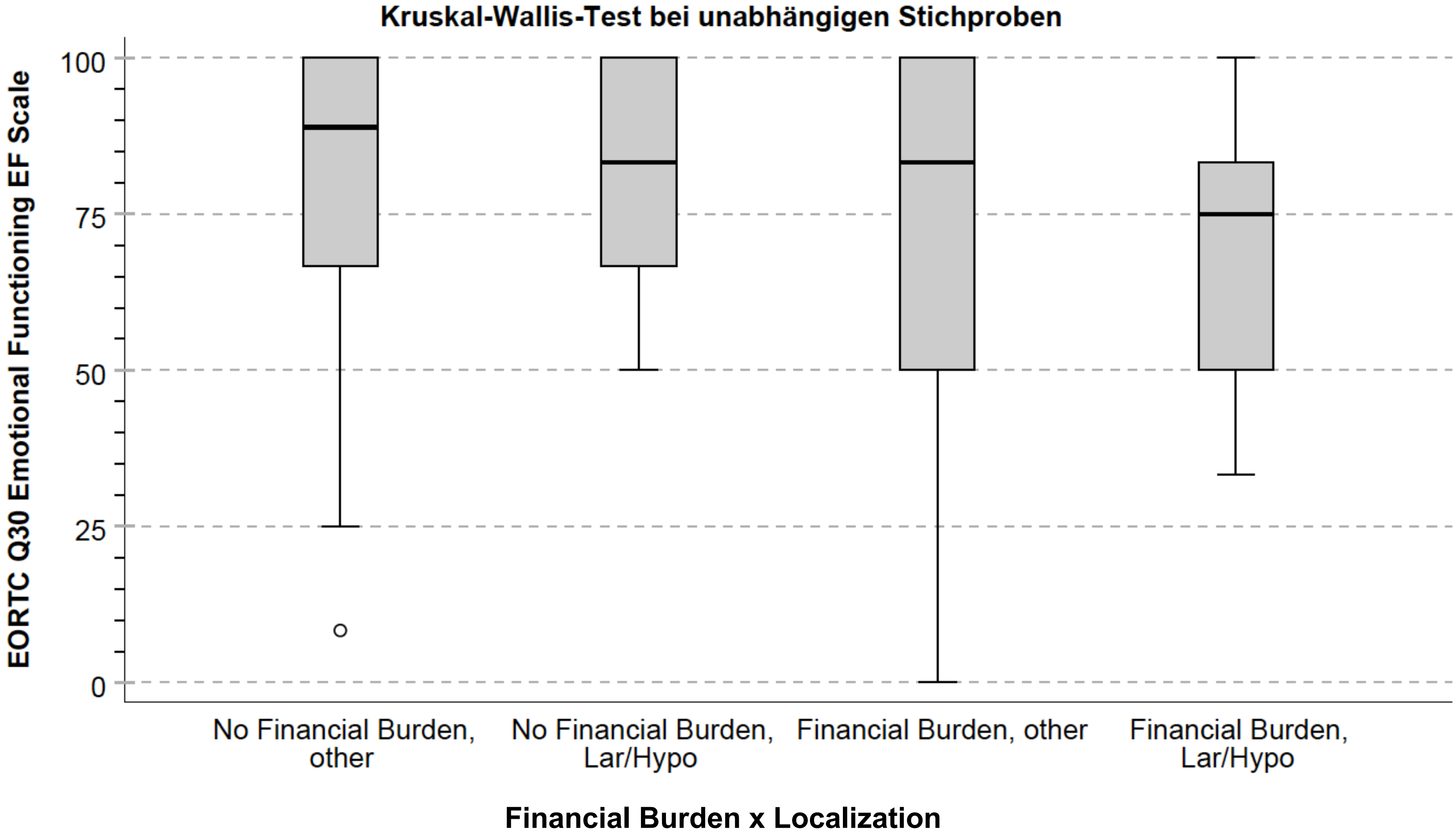
Kruskal-Wallis-Test bei unabhängigen Stichproben
EORTC Q30 Emotional Functioning EF Scale
100
75
50
25
0
No Financial Burden, other
No Financial Burden, Lar/Hypo
Financial Burden, other
Financial Burden, Lar/Hypo
Financial Burden x Localization

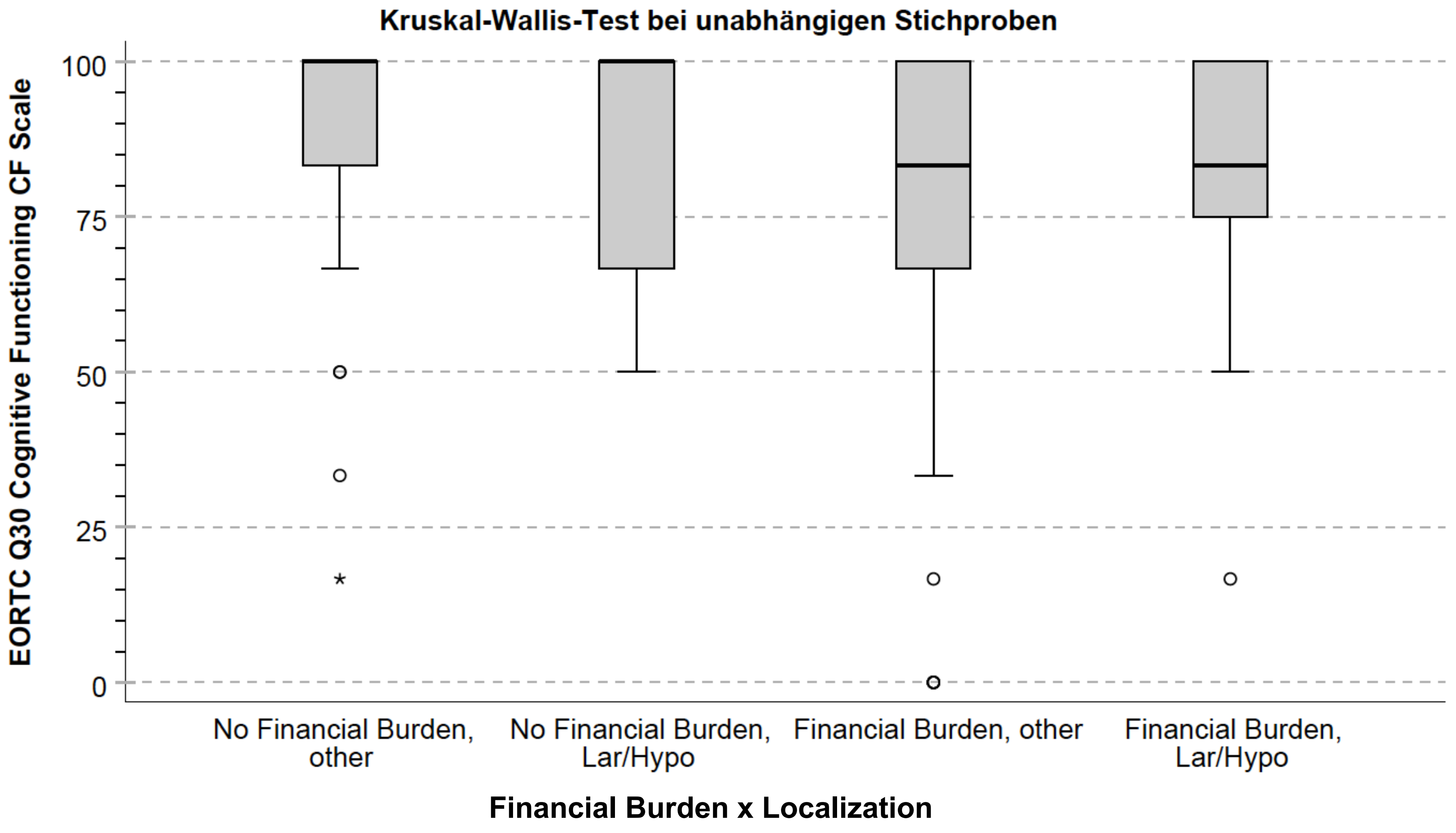
Kruskal-Wallis-Test bei unabhängigen Stichproben
EORTC Q30 Cognitive Functioning CF Scale
100
75
50
25
0
No Financial Burden, other
No Financial Burden, Lar/Hypo
Financial Burden, other
Financial Burden, Lar/Hypo
Financial Burden x Localization

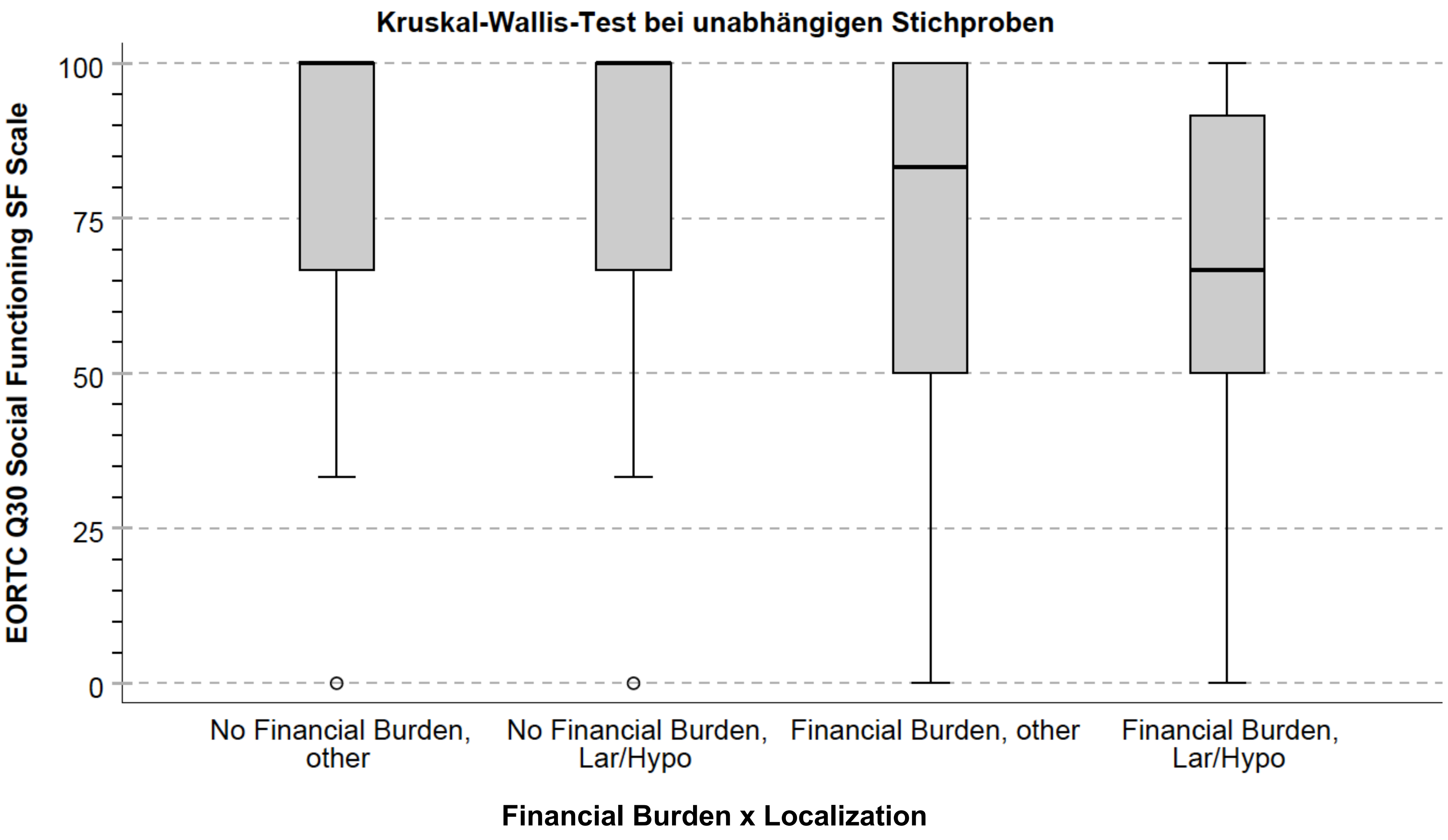
Kruskal-Wallis-Test bei unabhängigen Stichproben
EORTC Q30 Social Functioning SF Scale
100
75
50
25
0
No Financial Burden, other
No Financial Burden, Lar/Hypo
Financial Burden, other
Financial Burden, Lar/Hypo
Financial Burden x Localization

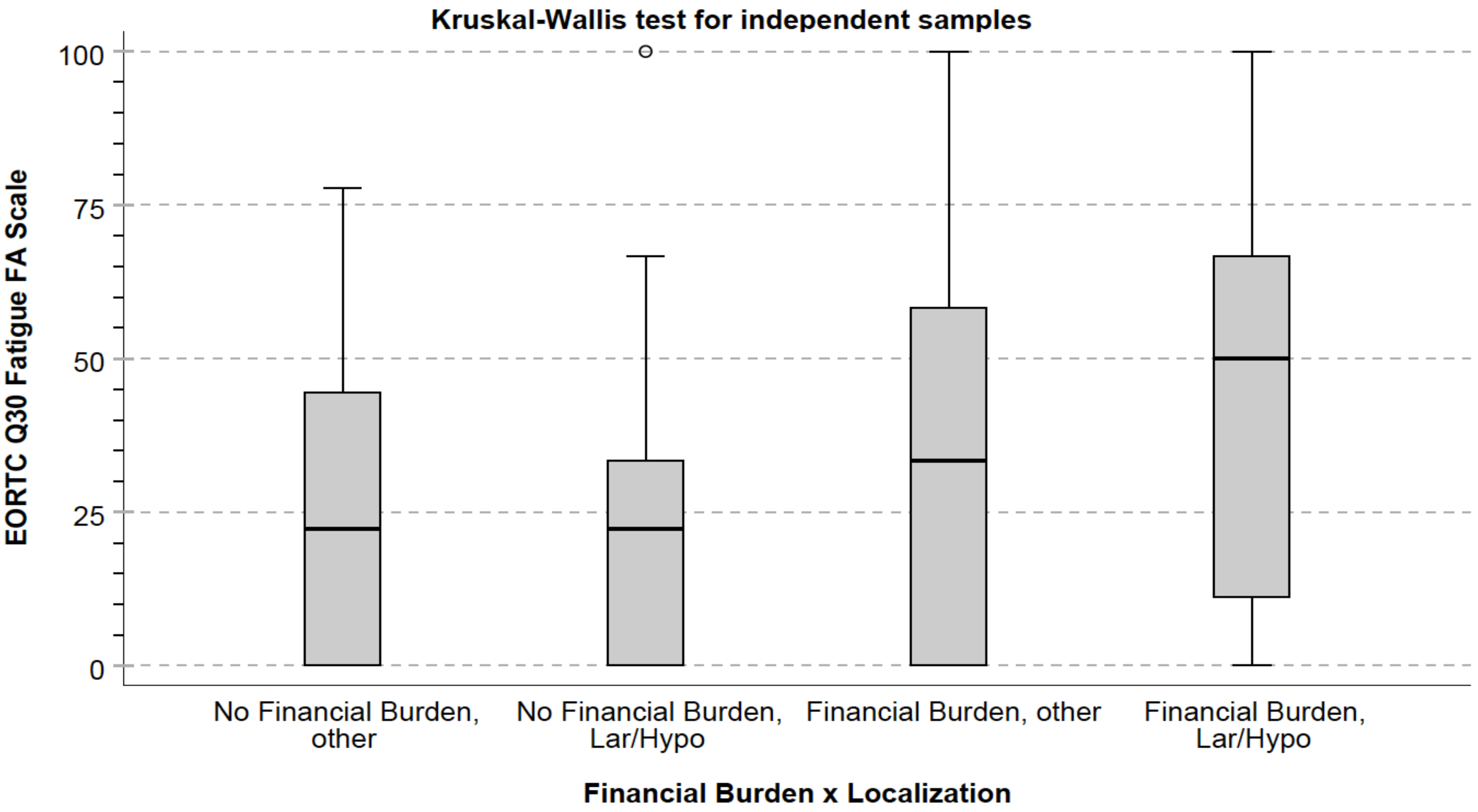

Kruskal-Wallis test for independent samples
EORTC Q30 Fatigue FA Scale
100
75
50
25
0
No Financial Burden, other
No Financial Burden, Lar/Hypo
Financial Burden, other
Financial Burden, Lar/Hypo
Financial Burden x Localization

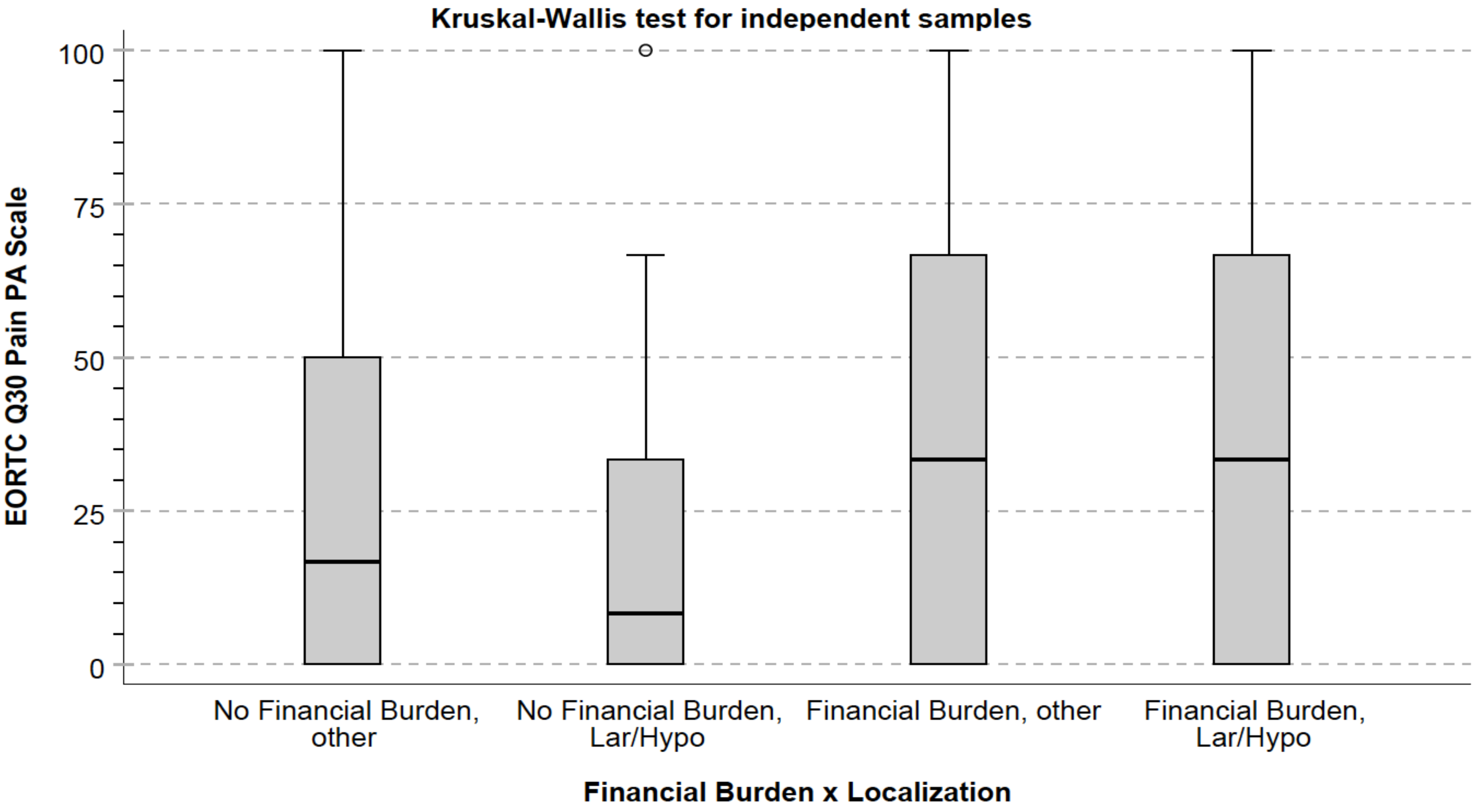

Kruskal-Wallis test for independent samples
EORTC Q30 Pain PA Scale
100
75
50
25
0
No Financial Burden, other
No Financial Burden, Lar/Hypo
Financial Burden, other
Financial Burden, Lar/Hypo
Financial Burden x Localization

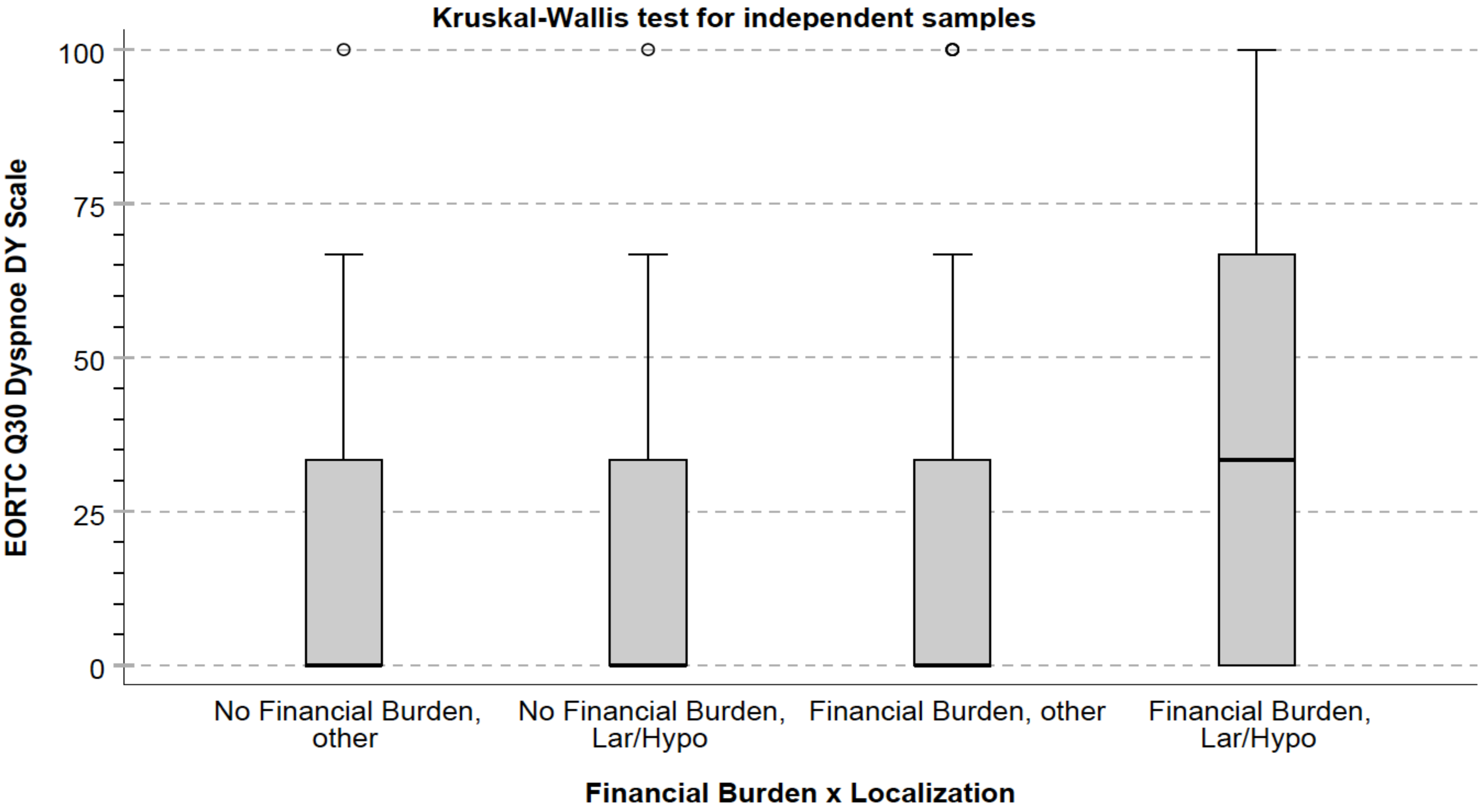
Kruskal-Wallis test for independent samples
EORTC Q30 Dyspnoe DY Scale
100
75
50
25
0
No Financial Burden, other
No Financial Burden, Lar/Hypo
Financial Burden, other
Financial Burden, Lar/Hypo
Financial Burden x Localization

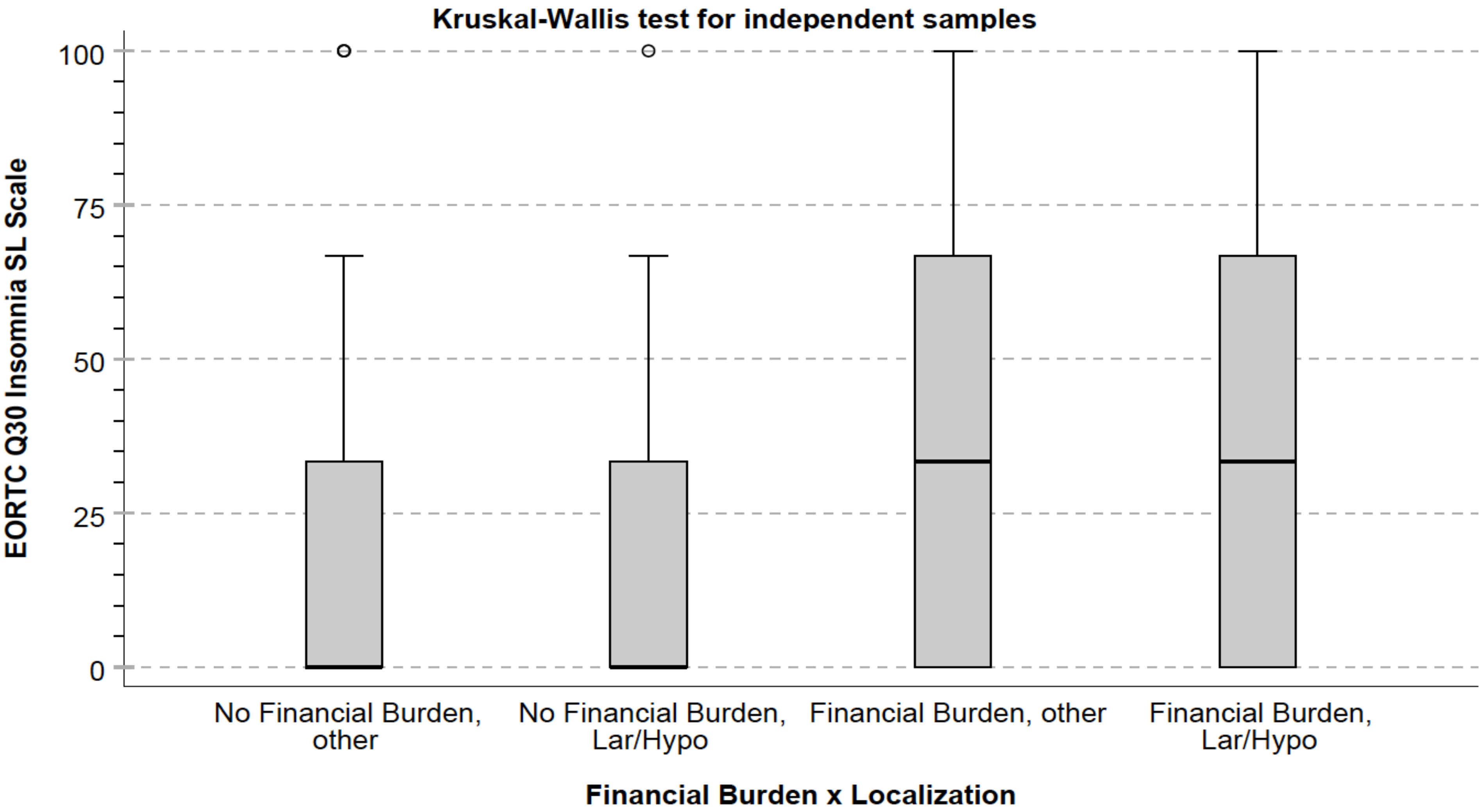
Kruskal-Wallis test for independent samples
EORTC Q30 Insomnia SL Scale
100
75
50
25
0
No Financial Burden, other
No Financial Burden, Lar/Hypo
Financial Burden, other
Financial Burden, Lar/Hypo
Financial Burden x Localization

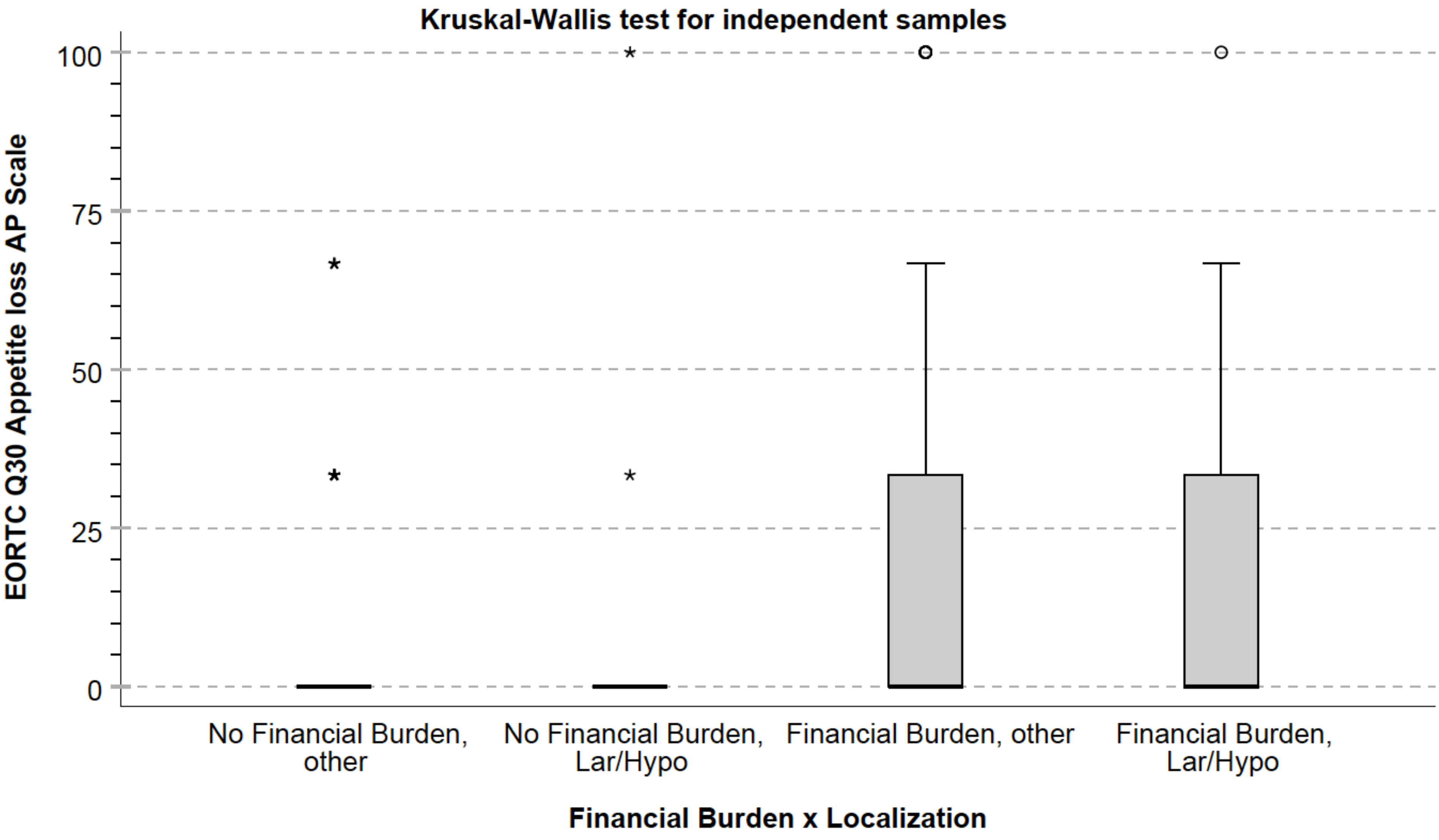
Kruskal-Wallis test for independent samples
EORTC Q30 Appetite loss AP Scale
100
75
50
25
0
No Financial Burden, other
No Financial Burden, Lar/Hypo
Financial Burden, other
Financial Burden, Lar/Hypo
Financial Burden x Localization

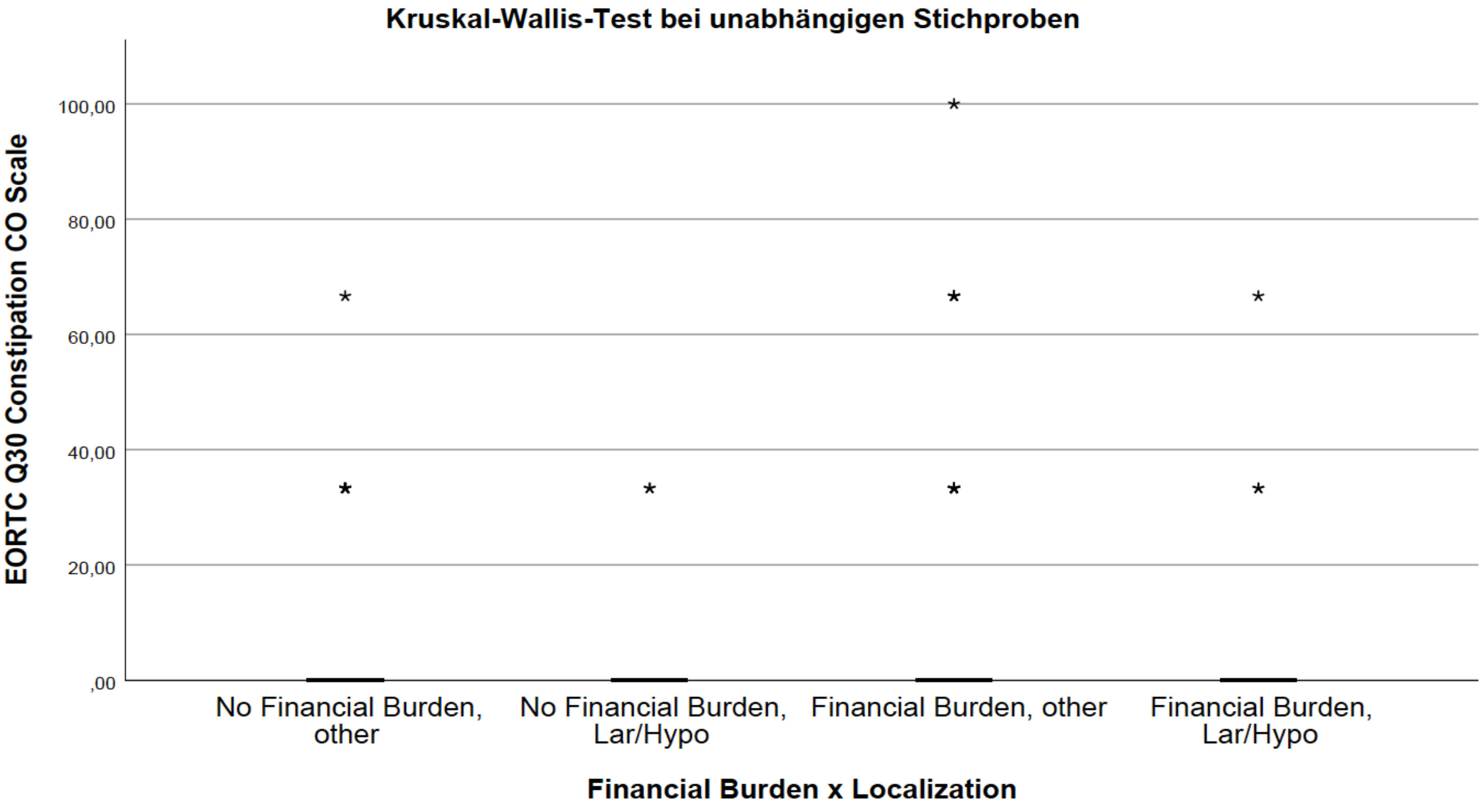
Kruskal-Wallis-Test bei unabhängigen Stichproben
EORTC Q30 Constipation CO Scale
100,00
80,00
60,00
40,00
20,00
,00
No Financial Burden, other
No Financial Burden, Lar/Hypo
Financial Burden, other
Financial Burden, Lar/Hypo
Financial Burden x Localization

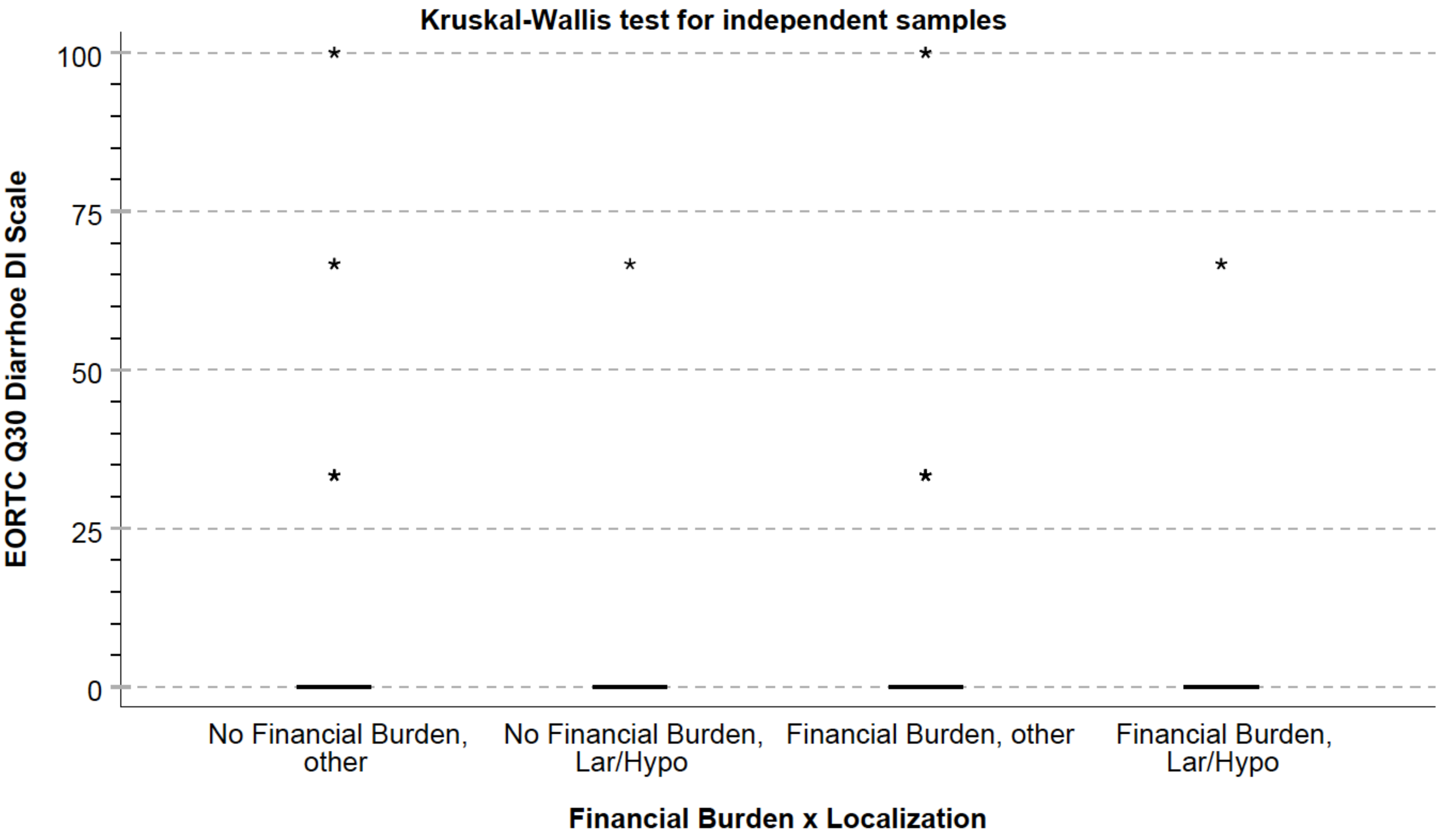
Kruskal-Wallis test for independent samples
EORTC Q30 Diarrhoe DI Scale
100
75
50
25
0
No Financial Burden, other
No Financial Burden, Lar/Hypo
Financial Burden, other
Financial Burden, Lar/Hypo
Financial Burden x Localization

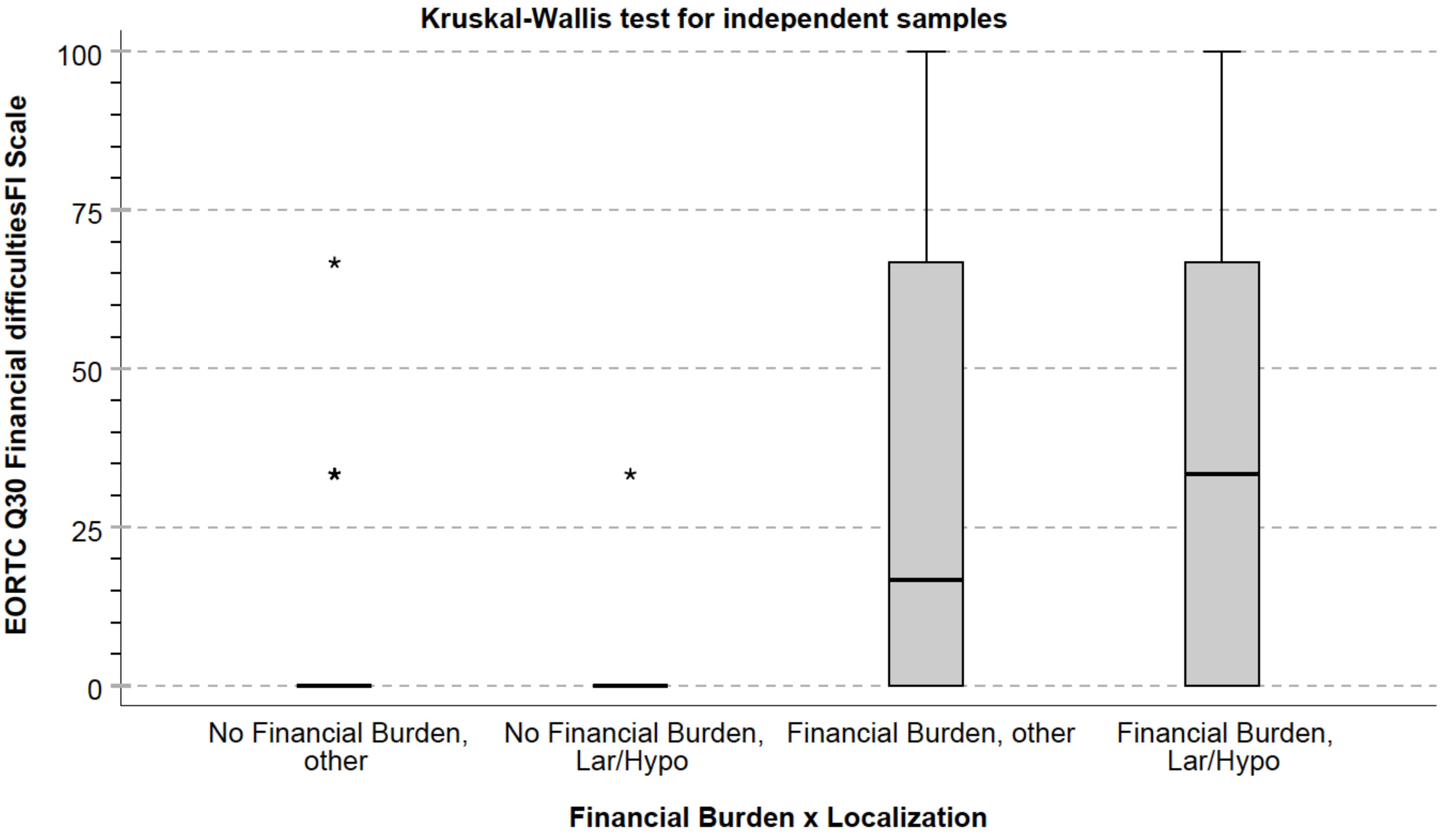
Kruskal-Wallis test for independent samples
EORTC Q30 Financial difficultiesFI Scale
100
75
50
25
0
No Financial Burden, other
No Financial Burden, Lar/Hypo
Financial Burden, other
Financial Burden, Lar/Hypo
Financial Burden x Localization
