# Supplementary material for: The impact of financial burden on quality of life among German head and neck cancer survivors
Source: BMC Cancer. 2025 Mar 20;25:514. doi: 10.1186/s12885-025-13927-1 (PMC11927114; doi:10.1186/s12885-025-13927-1)
Supplement: Supplementary file 2 — Supplementary Material 2 [file 12885_2025_13927_MOESM2_ESM.pdf]

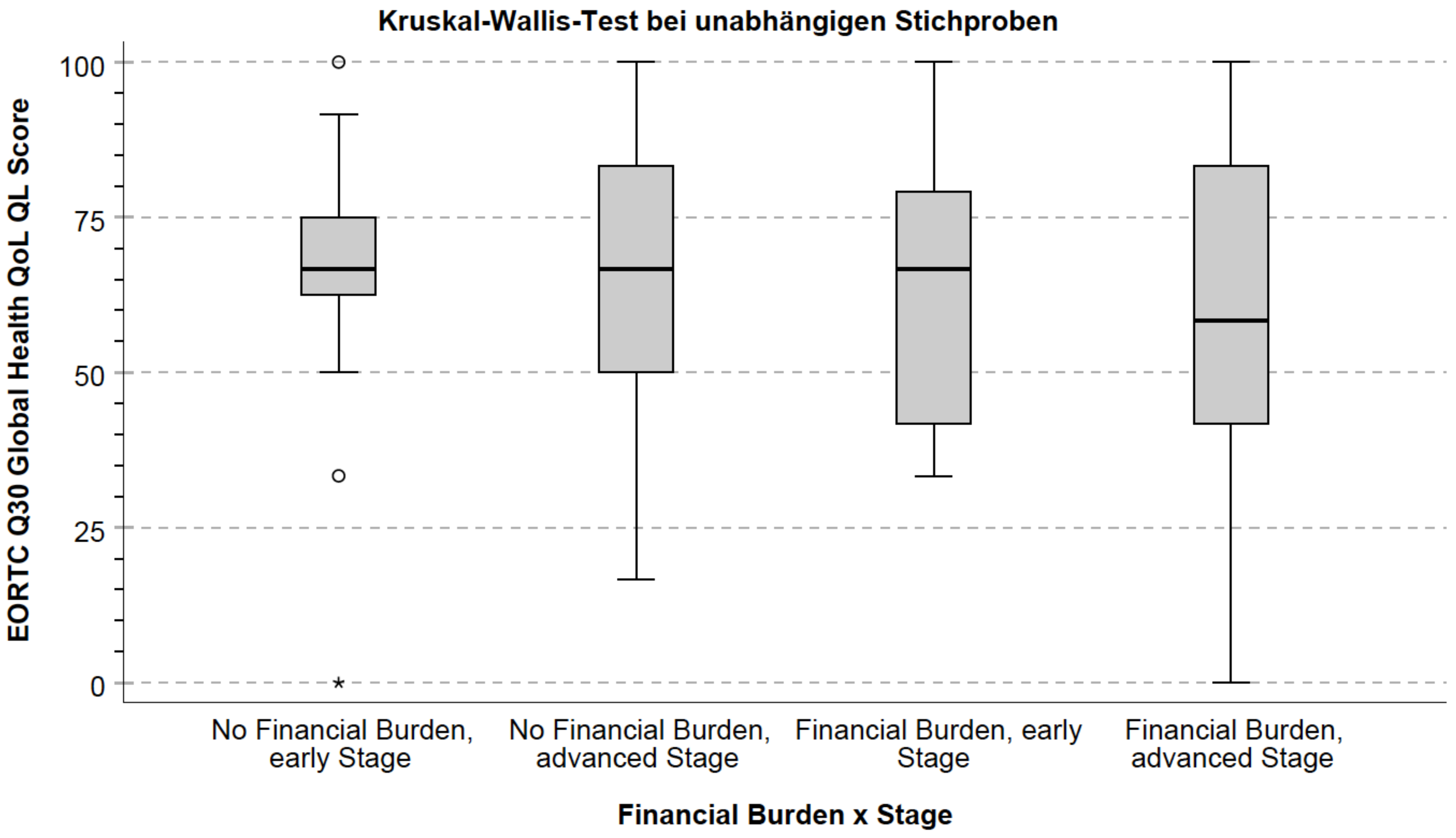

Kruskal-Wallis-Test bei unabhängigen Stichproben
EORTC Q30 Global Health QoL QL Score
100
75
50
25
0
No Financial Burden, early Stage
No Financial Burden, advanced Stage
Financial Burden, early Stage
Financial Burden, advanced Stage
Financial Burden x Stage

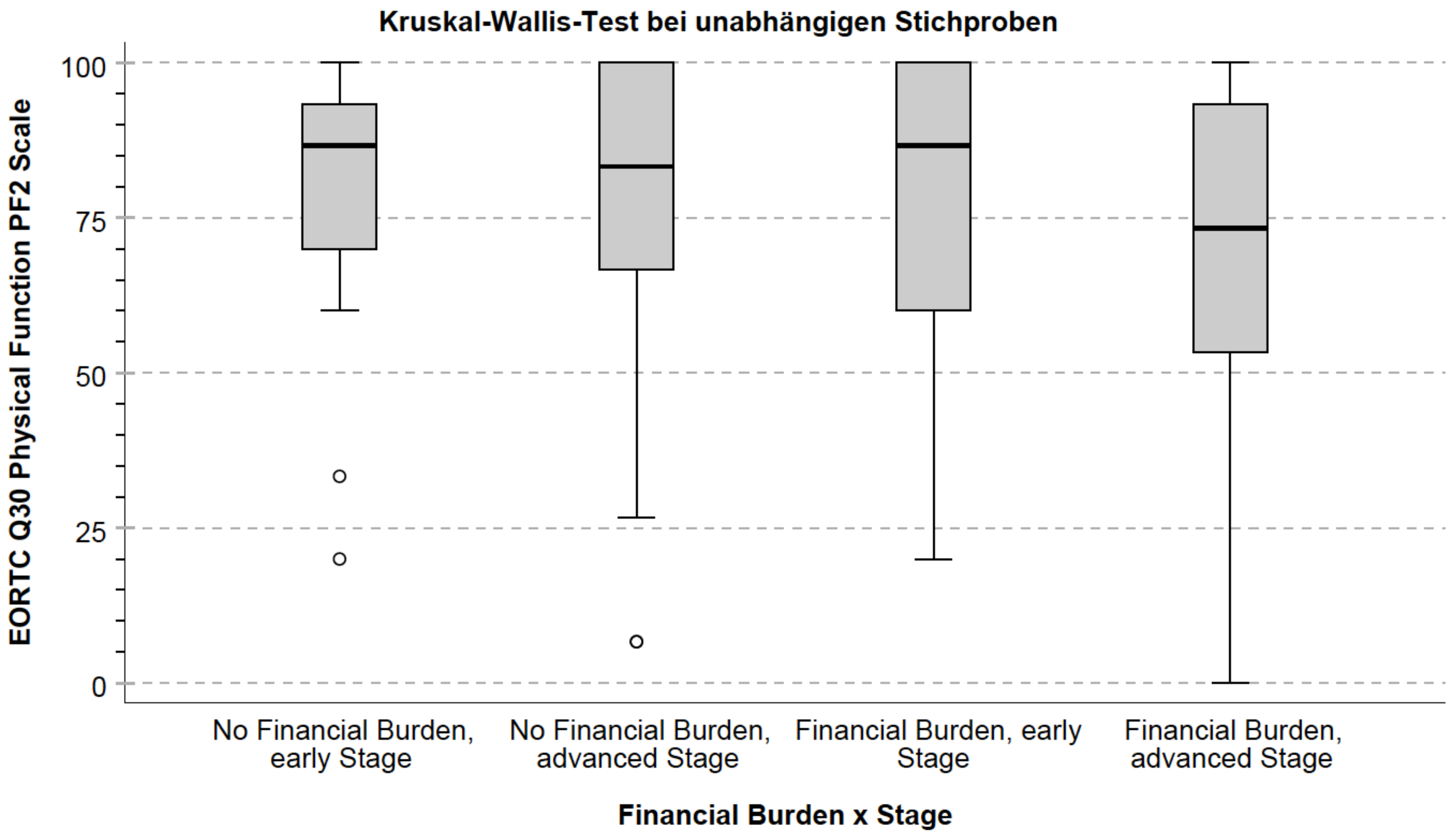

Kruskal-Wallis-Test bei unabhängigen Stichproben
EORTC Q30 Physical Function PF2 Scale
100
75
50
25
0
No Financial Burden, early Stage
No Financial Burden, advanced Stage
Financial Burden, early Stage
Financial Burden, advanced Stage
Financial Burden x Stage

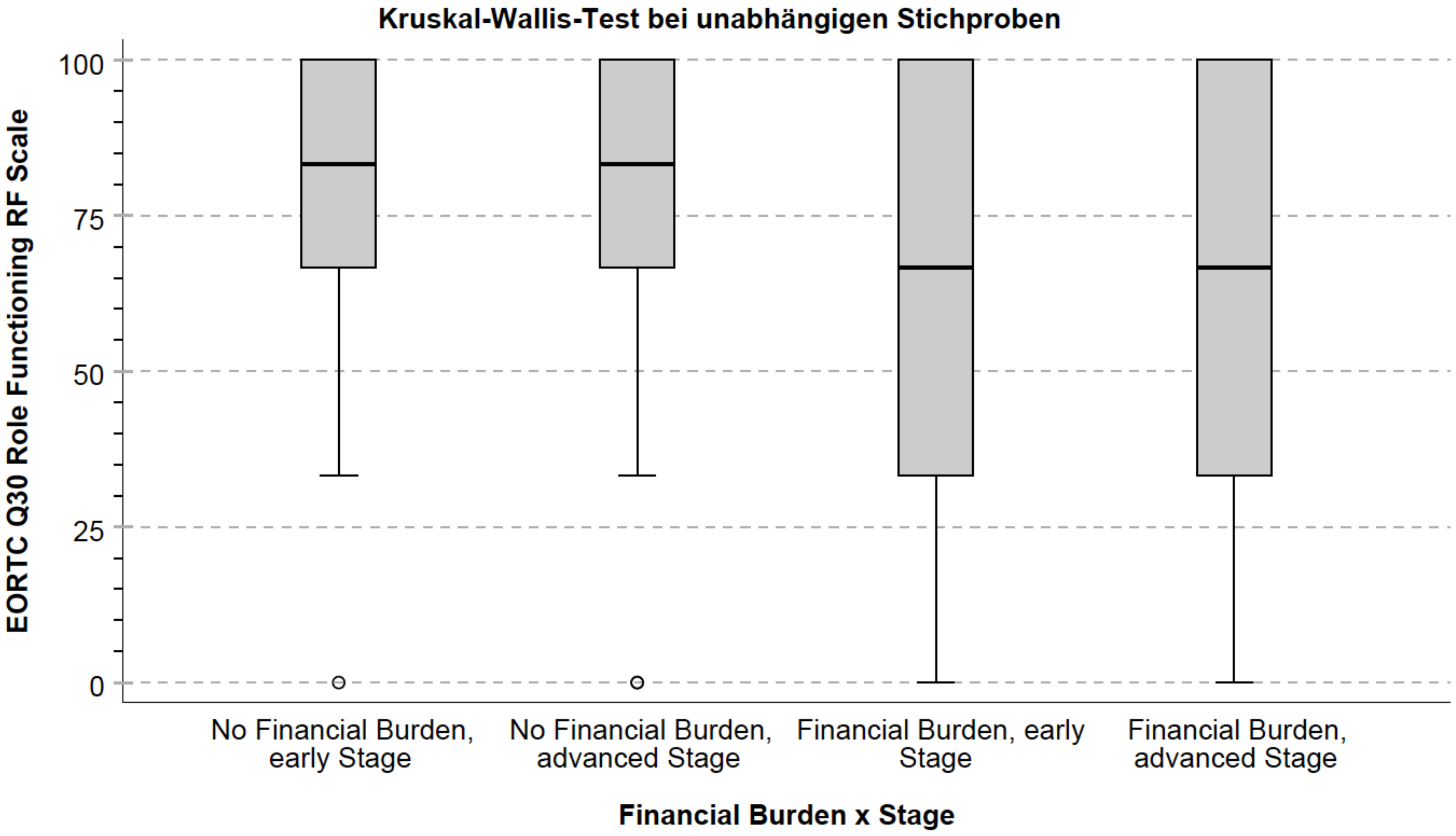
Kruskal-Wallis-Test bei unabhängigen Stichproben
EORTC Q30 Role Functioning RF Scale
100
75
50
25
0
No Financial Burden, early Stage
No Financial Burden, advanced Stage
Financial Burden, early Stage
Financial Burden, advanced Stage
Financial Burden x Stage

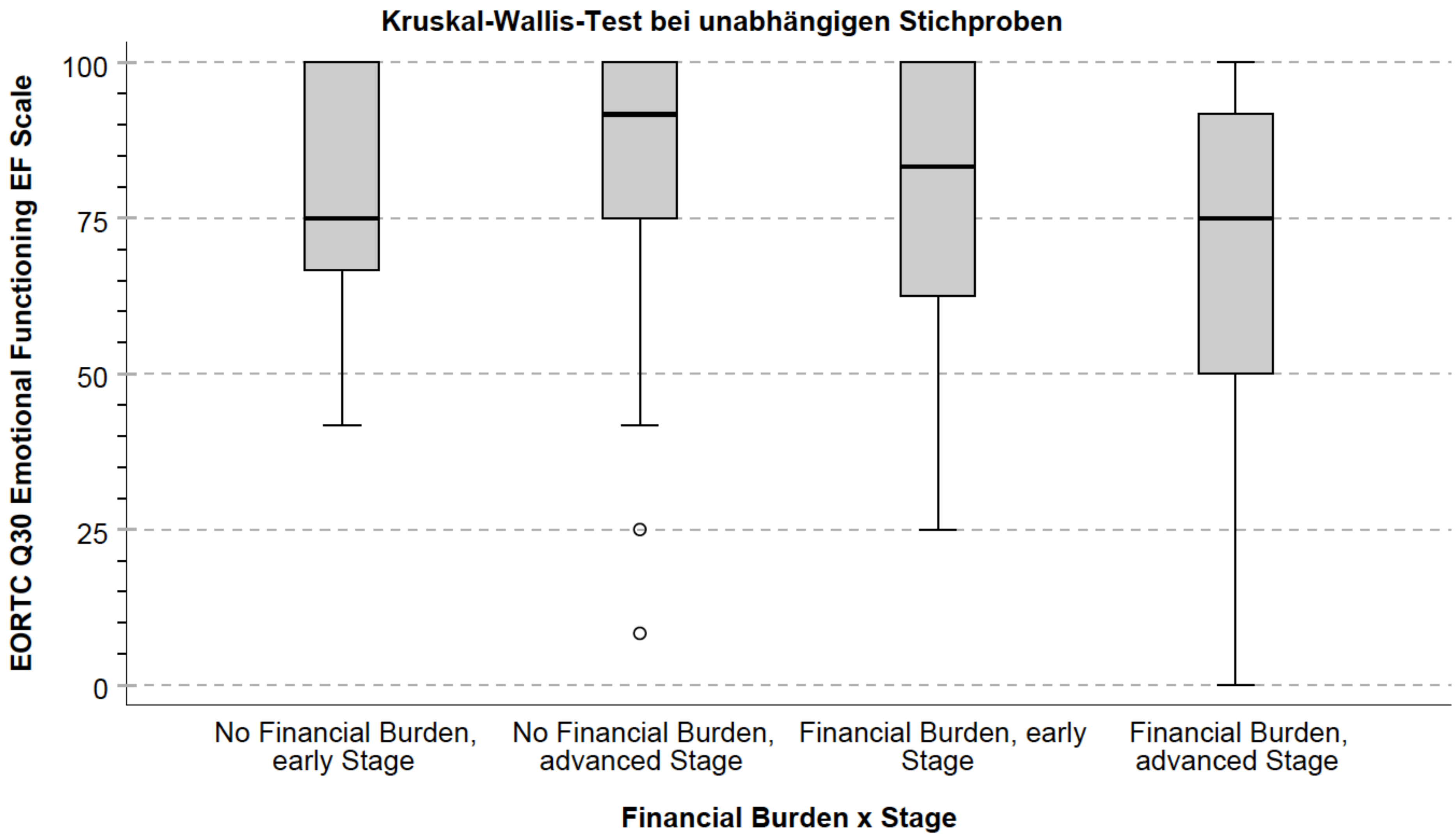
Kruskal-Wallis-Test bei unabhängigen Stichproben
EORTC Q30 Emotional Functioning EF Scale
100
75
50
25
0
No Financial Burden, early Stage
No Financial Burden, advanced Stage
Financial Burden, early Stage
Financial Burden, advanced Stage
Financial Burden x Stage

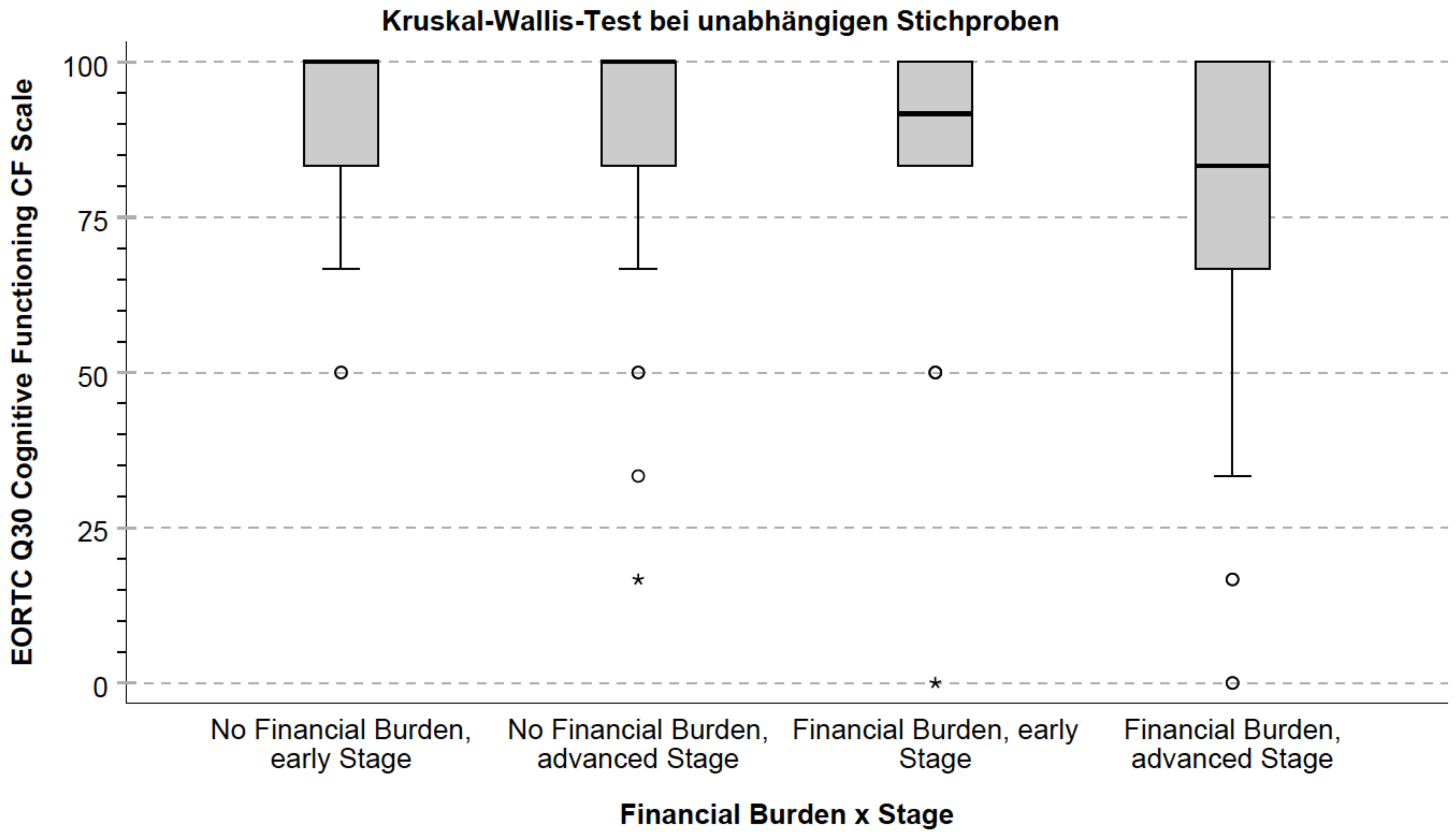
Kruskal-Wallis-Test bei unabhängigen Stichproben
EORTC Q30 Cognitive Functioning CF Scale
100
75
50
25
0
No Financial Burden, early Stage
No Financial Burden, advanced Stage
Financial Burden, early Stage
Financial Burden, advanced Stage
Financial Burden x Stage

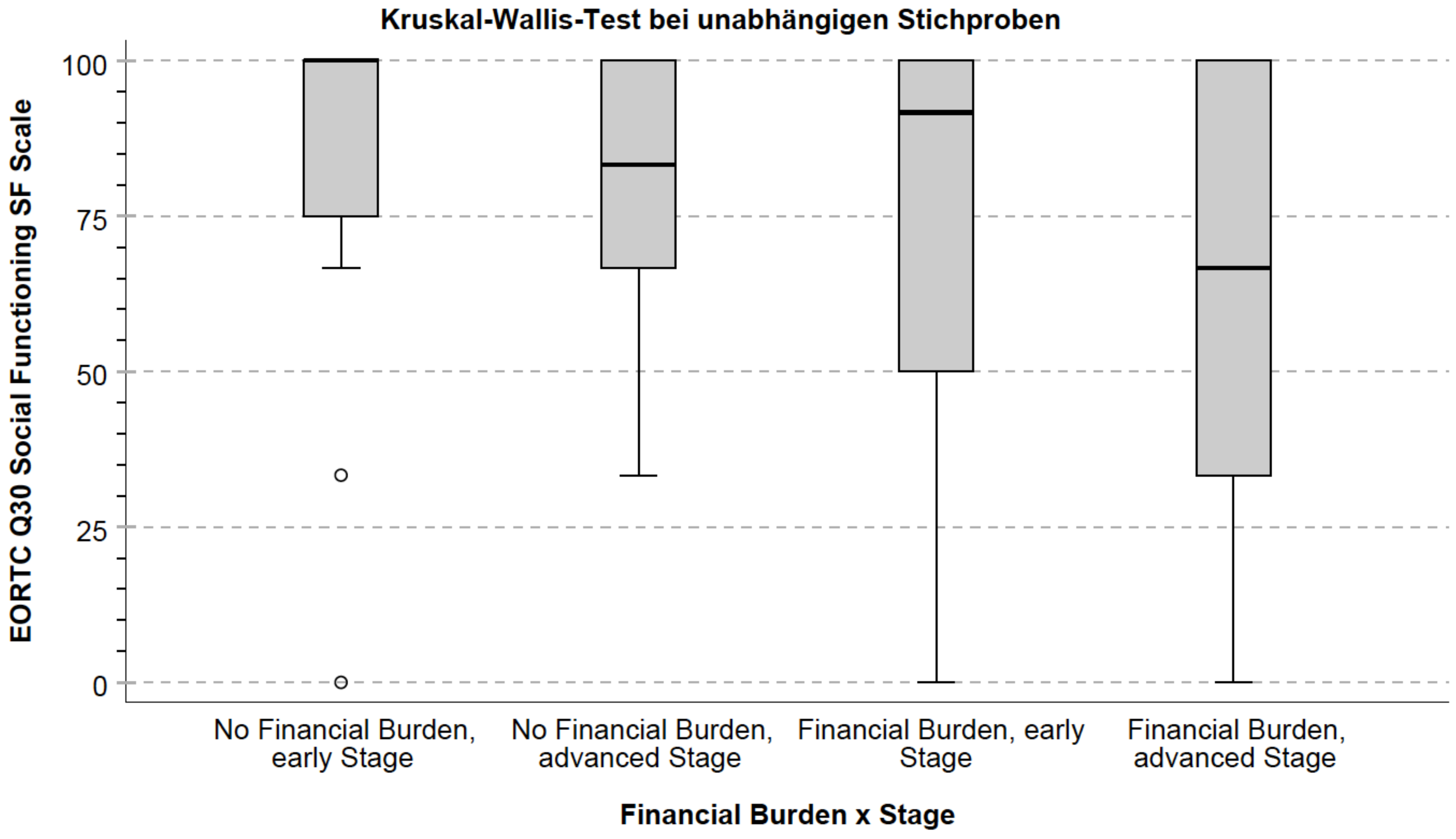
Kruskal-Wallis-Test bei unabhängigen Stichproben
EORTC Q30 Social Functioning SF Scale
100
75
50
25
0
No Financial Burden, early Stage
No Financial Burden, advanced Stage
Financial Burden, early Stage
Financial Burden, advanced Stage
Financial Burden x Stage

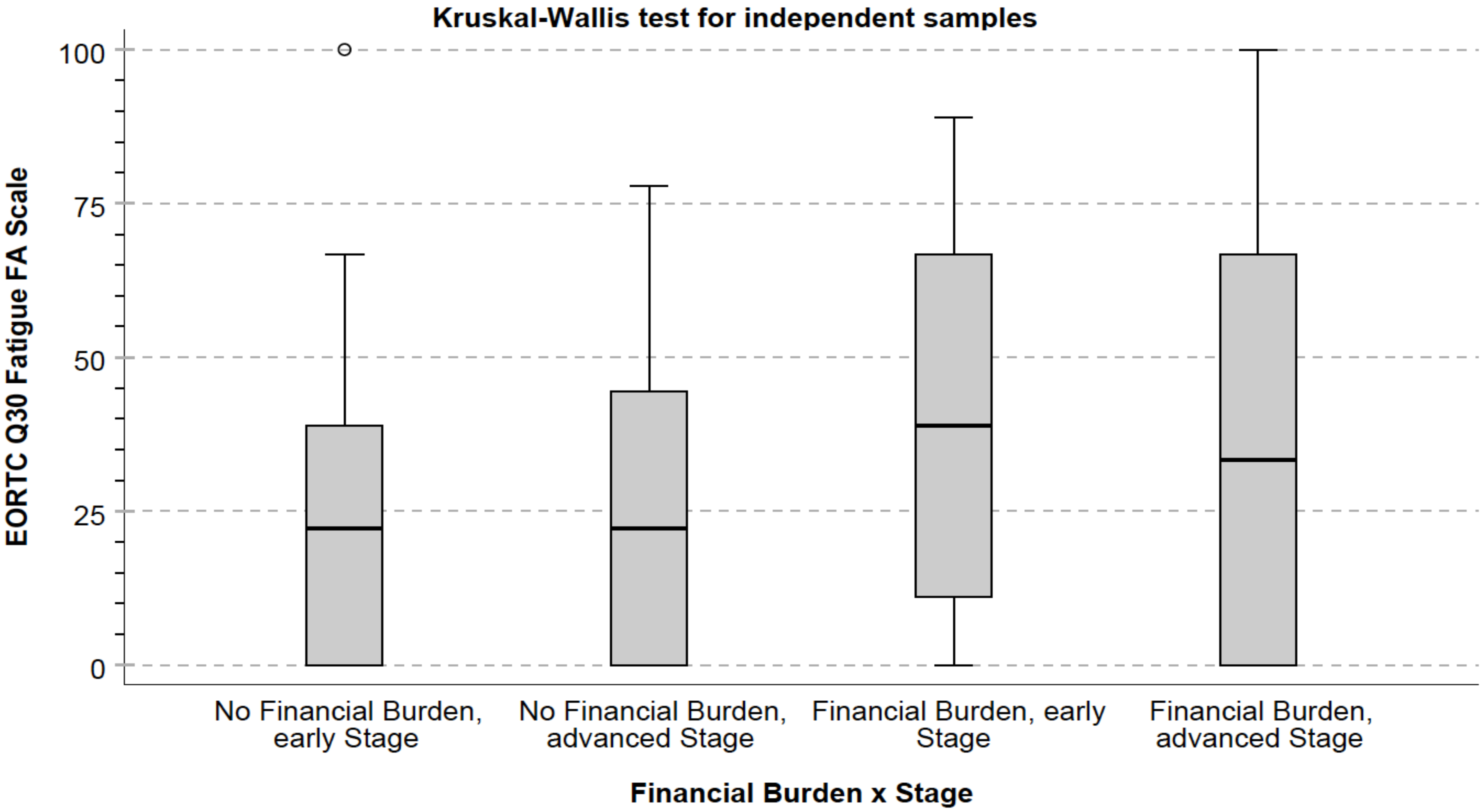
Kruskal-Wallis test for independent samples
EORTC Q30 Fatigue FA Scale
100
75
50
25
0
No Financial Burden, early Stage
No Financial Burden, advanced Stage
Financial Burden, early Stage
Financial Burden, advanced Stage
Financial Burden x Stage

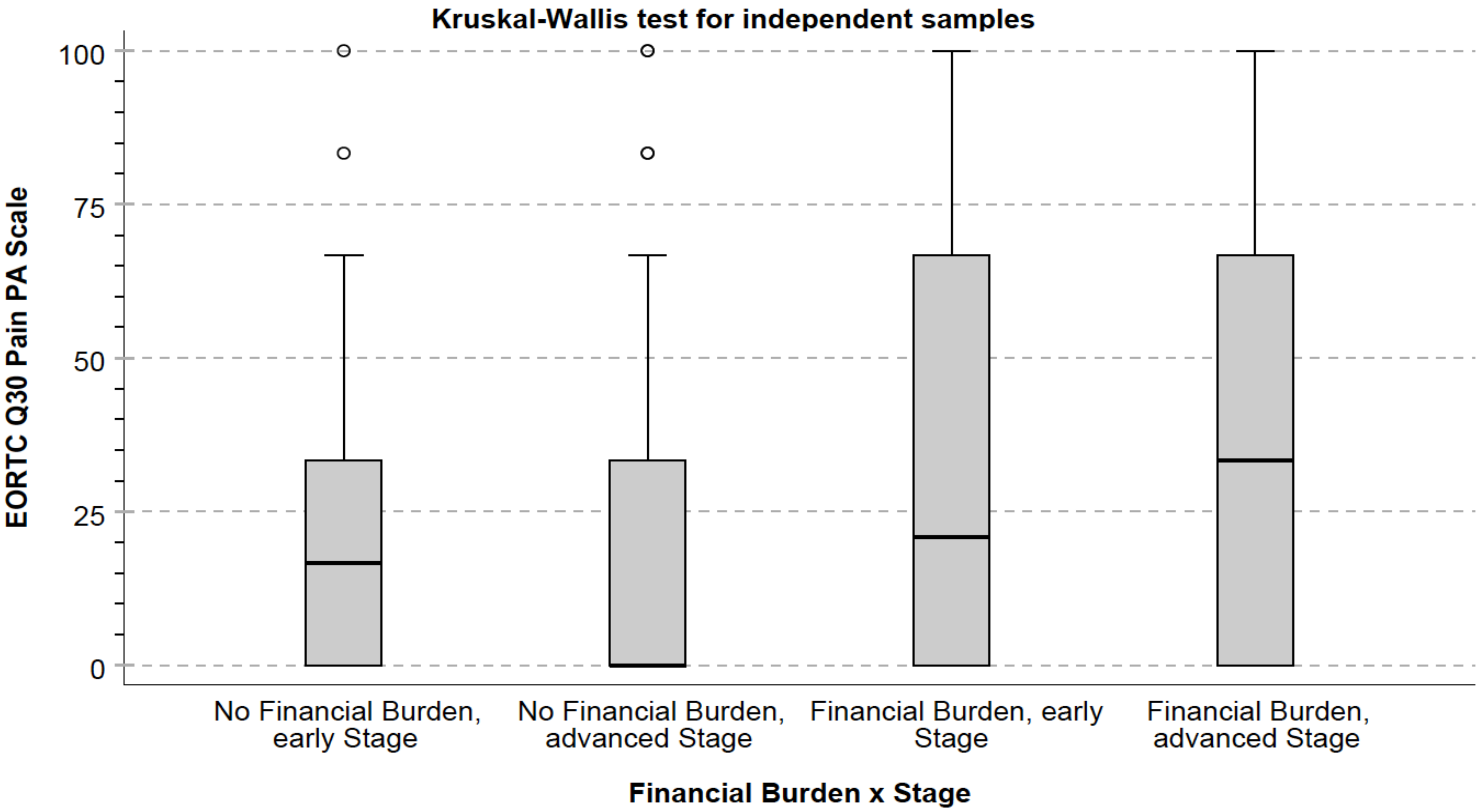
Kruskal-Wallis test for independent samples
EORTC Q30 Pain PA Scale
100
75
50
25
0
No Financial Burden, early Stage
No Financial Burden, advanced Stage
Financial Burden, early Stage
Financial Burden, advanced Stage
Financial Burden x Stage

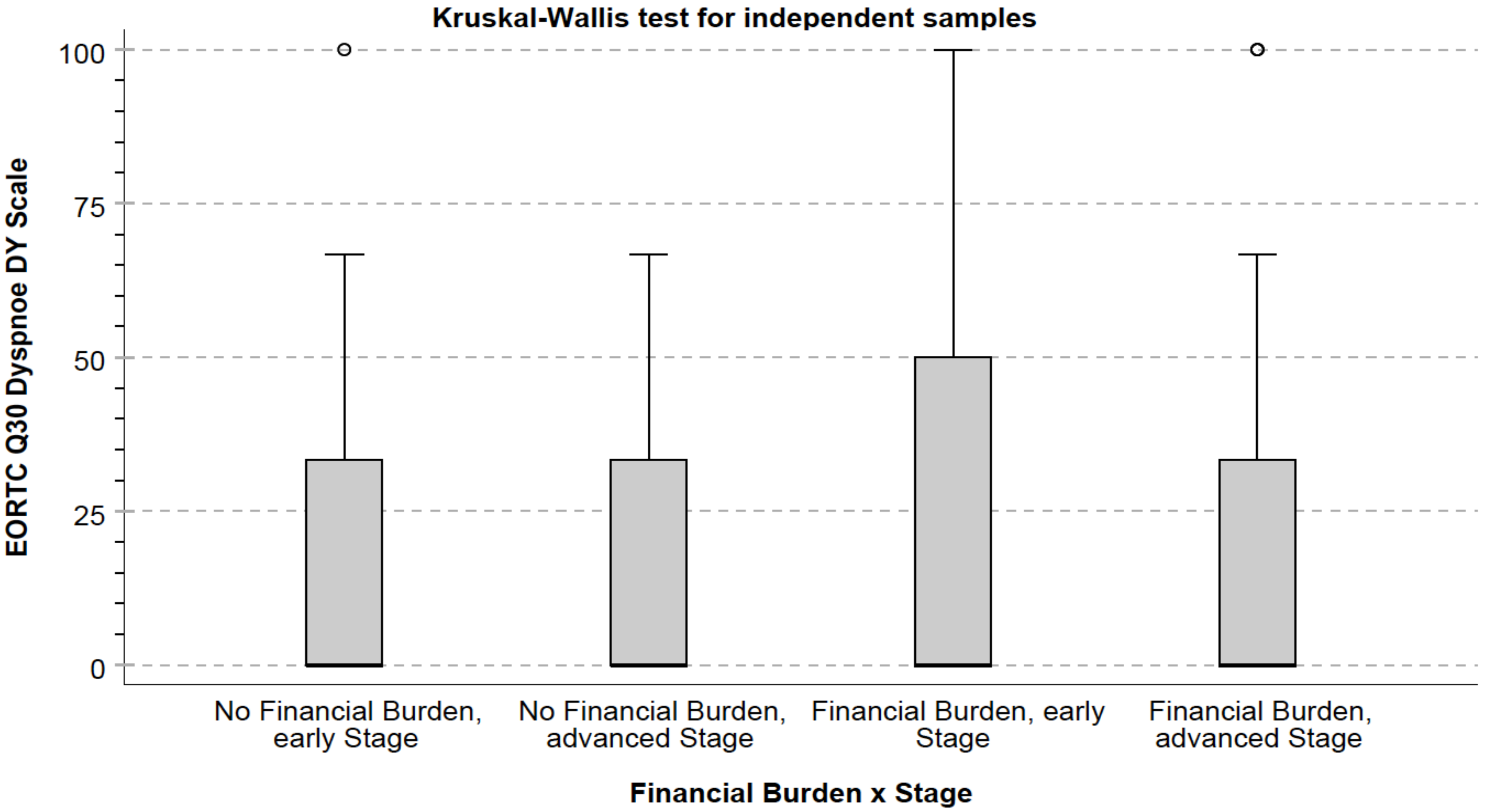
Kruskal-Wallis test for independent samples
EORTC Q30 Dyspnoe DY Scale
100
75
50
25
0
No Financial Burden, early Stage
No Financial Burden, advanced Stage
Financial Burden, early Stage
Financial Burden, advanced Stage
Financial Burden x Stage

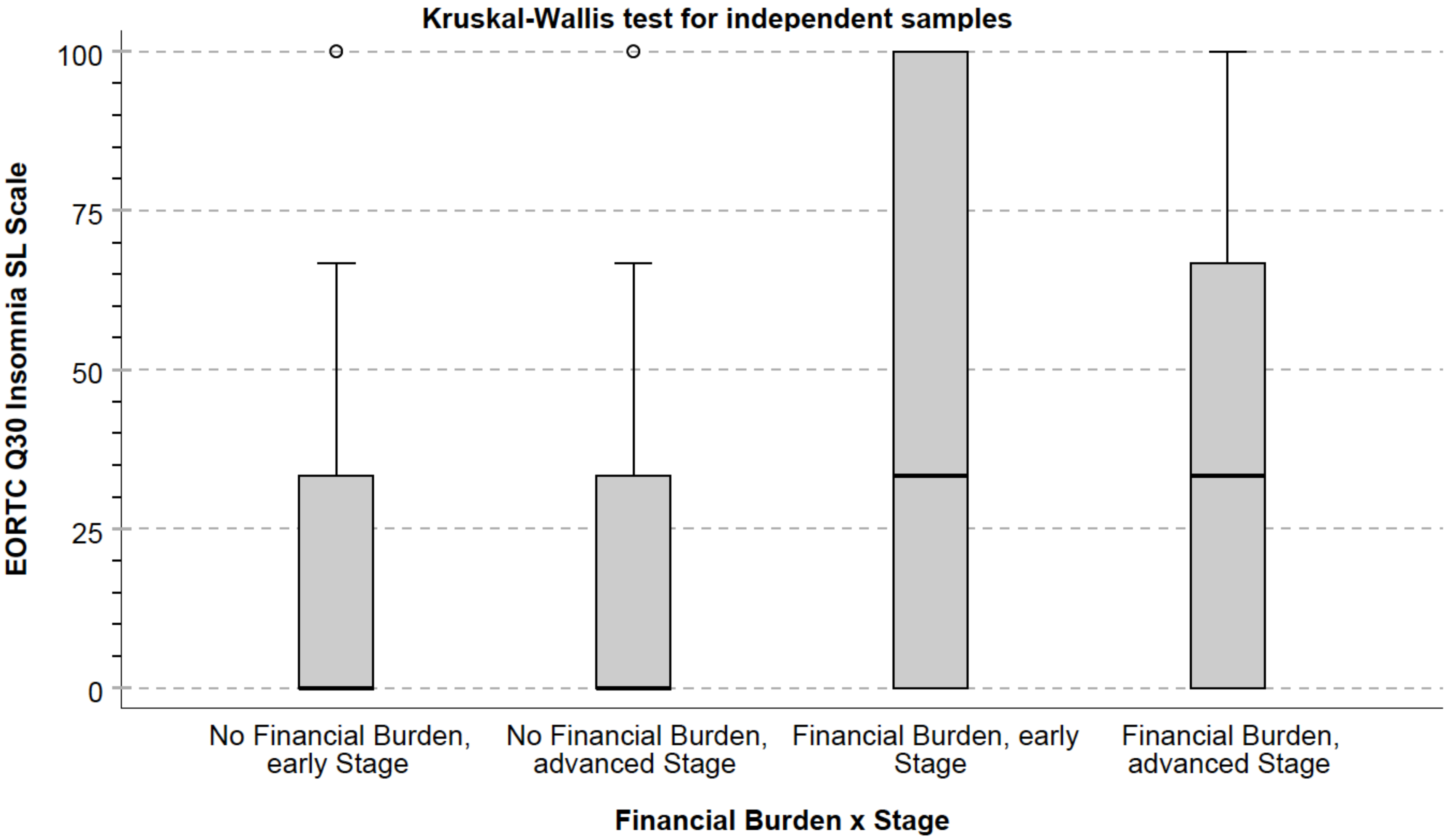
Kruskal-Wallis test for independent samples
EORTC Q30 Insomnia SL Scale
100
75
50
25
0
No Financial Burden, early Stage
No Financial Burden, advanced Stage
Financial Burden, early Stage
Financial Burden, advanced Stage
Financial Burden x Stage

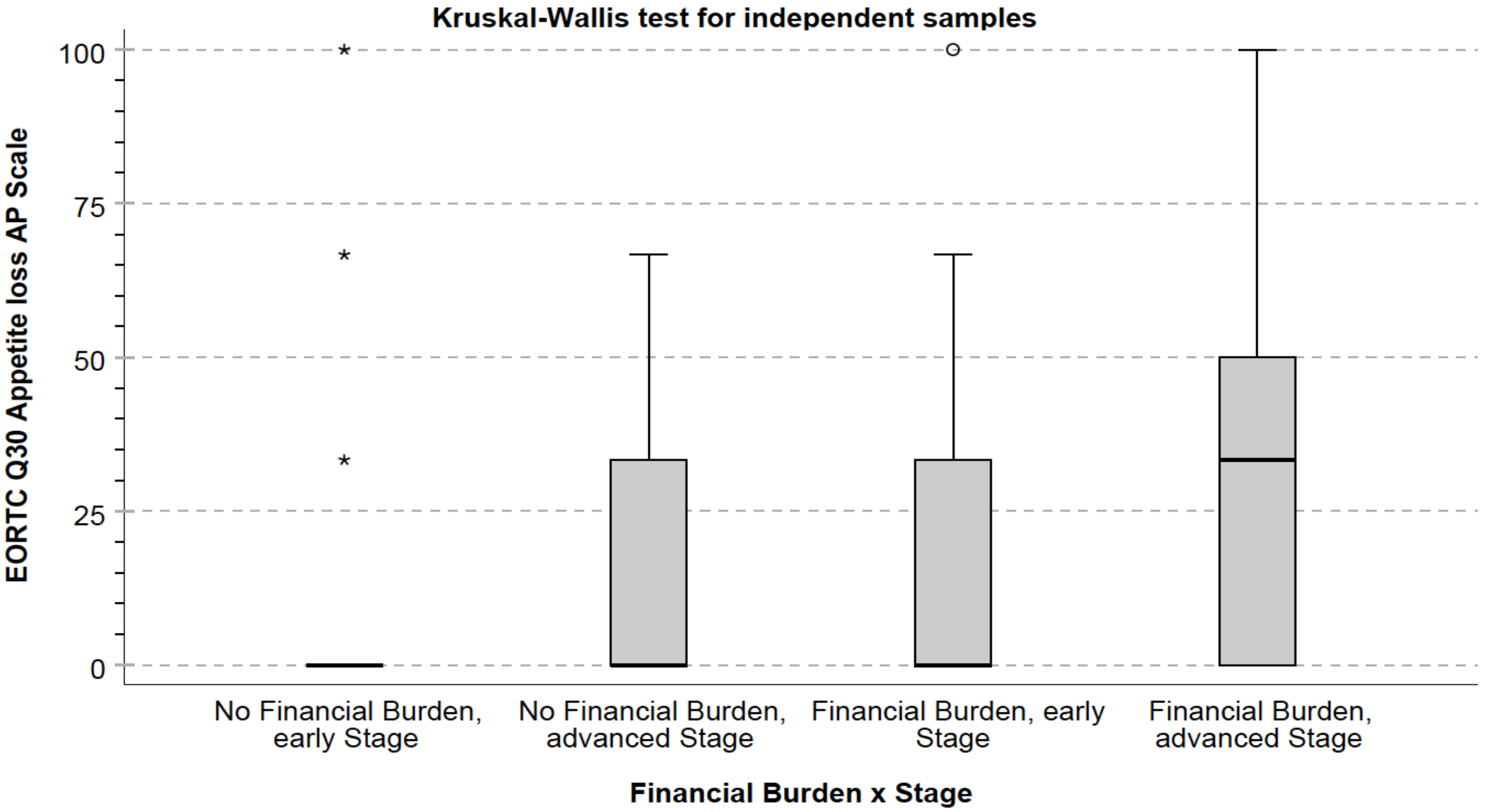
Kruskal-Wallis test for independent samples
EORTC Q30 Appetite loss AP Scale
100
75
50
25
0
No Financial Burden, early Stage
No Financial Burden, advanced Stage
Financial Burden, early Stage
Financial Burden, advanced Stage
Financial Burden x Stage

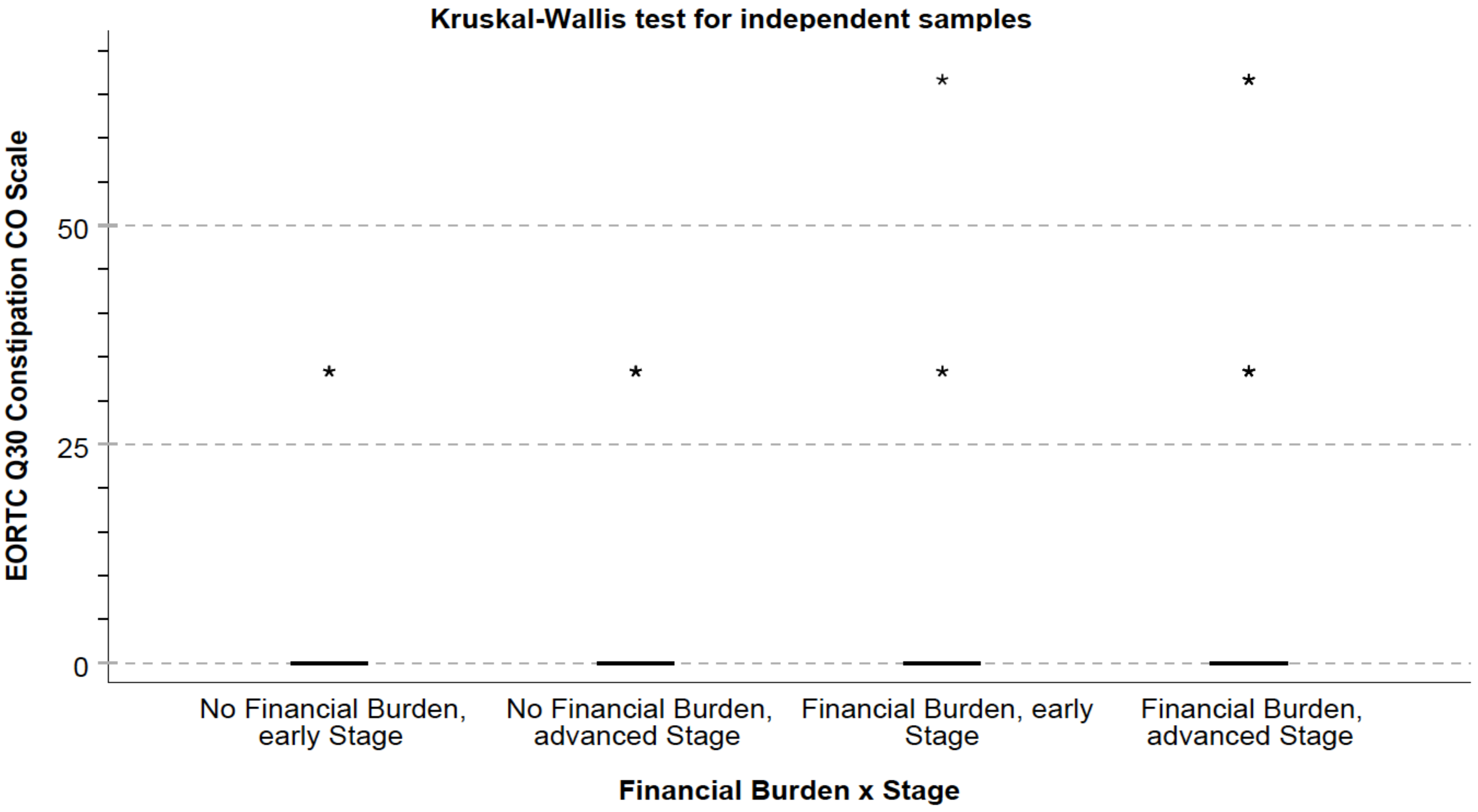
Kruskal-Wallis test for independent samples
EORTC Q30 Constipation CO Scale
50
25
0
No Financial Burden, early Stage
No Financial Burden, advanced Stage
Financial Burden, early Stage
Financial Burden, advanced Stage
Financial Burden x Stage

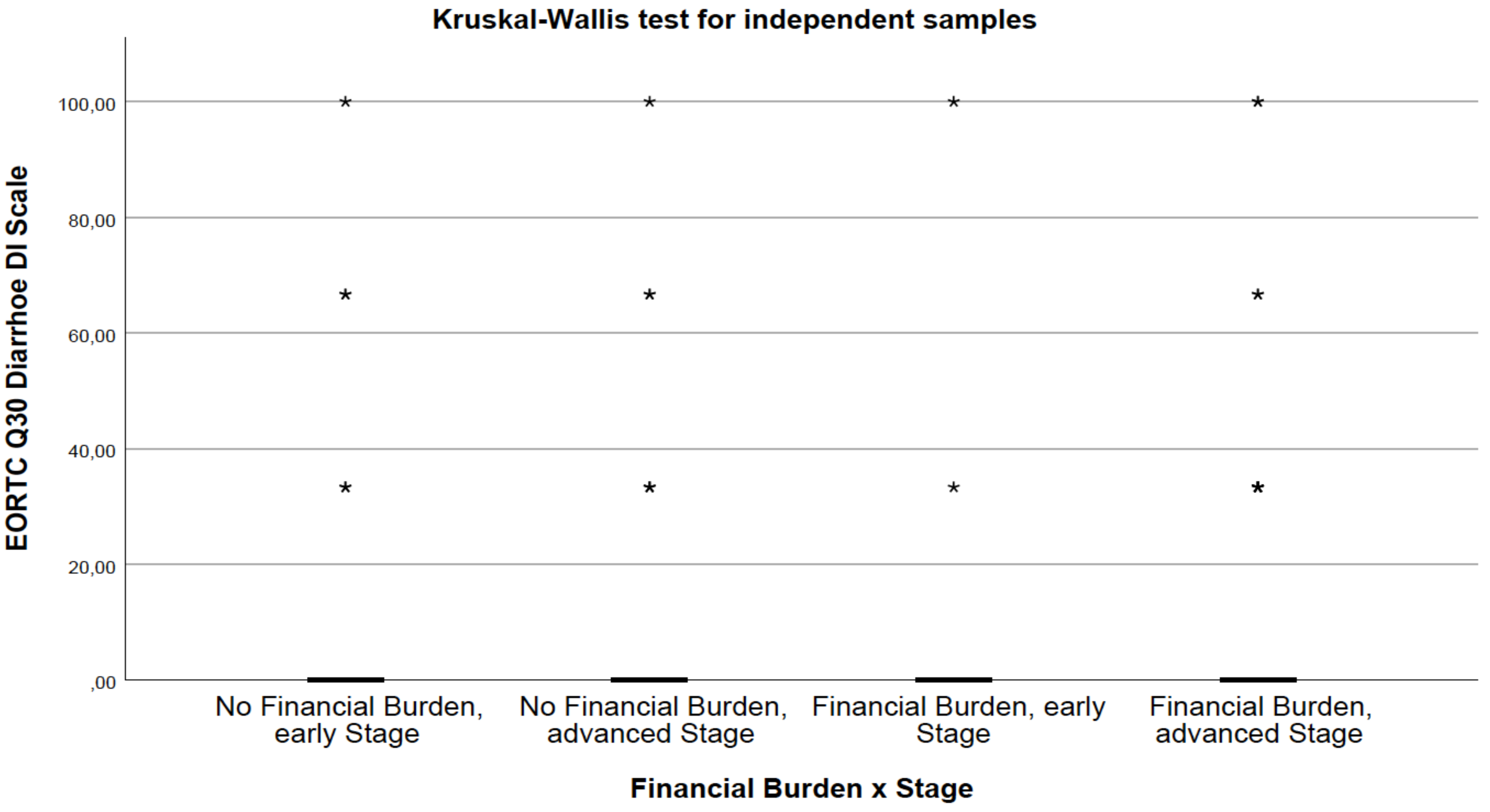
Kruskal-Wallis test for independent samples
EORTC Q30 Diarrhoe DI Scale
100,00
80,00
60,00
40,00
20,00
,00
No Financial Burden, early Stage
No Financial Burden, advanced Stage
Financial Burden, early Stage
Financial Burden, advanced Stage
Financial Burden x Stage

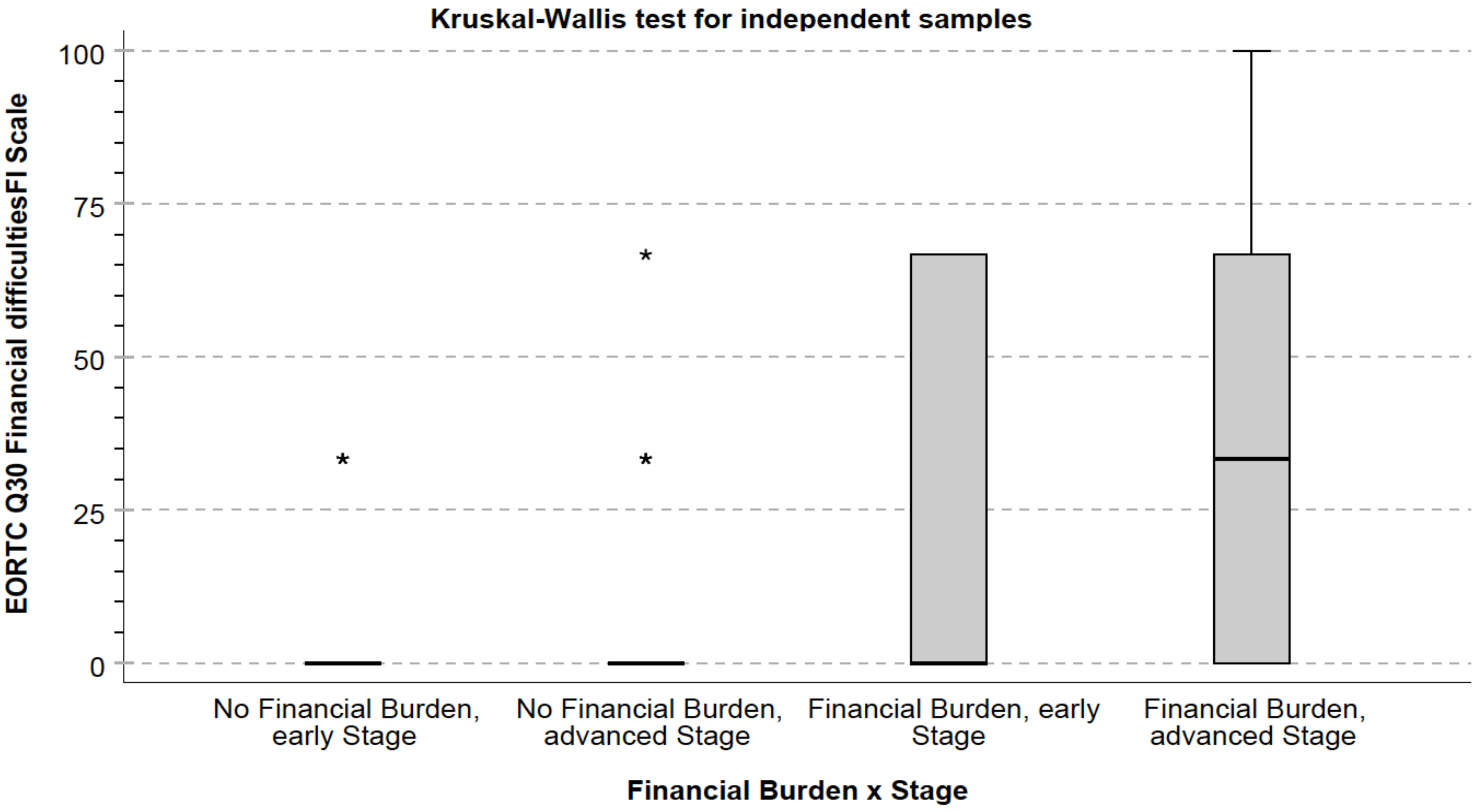
Kruskal-Wallis test for independent samples
EORTC Q30 Financial difficultiesFI Scale
100
75
50
25
0
No Financial Burden, early Stage
No Financial Burden, advanced Stage
Financial Burden, early Stage
Financial Burden, advanced Stage
Financial Burden x Stage
