# Supplementary figures and images for: The impact of financial burden on quality of life among German head and neck cancer survivors
Source: BMC Cancer. 2025 Mar 20;25:514. doi: 10.1186/s12885-025-13927-1 (PMC11927114; doi:10.1186/s12885-025-13927-1)

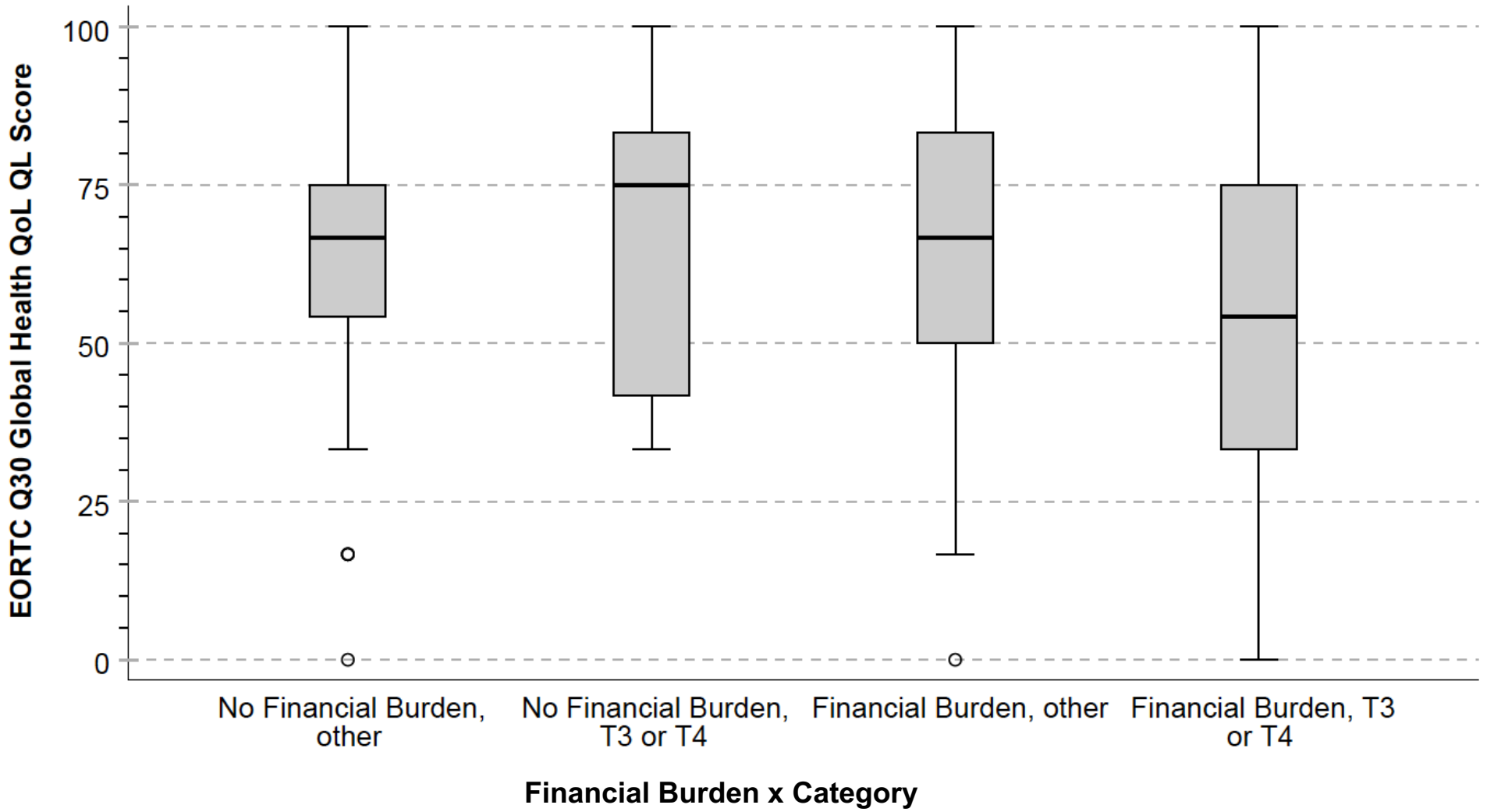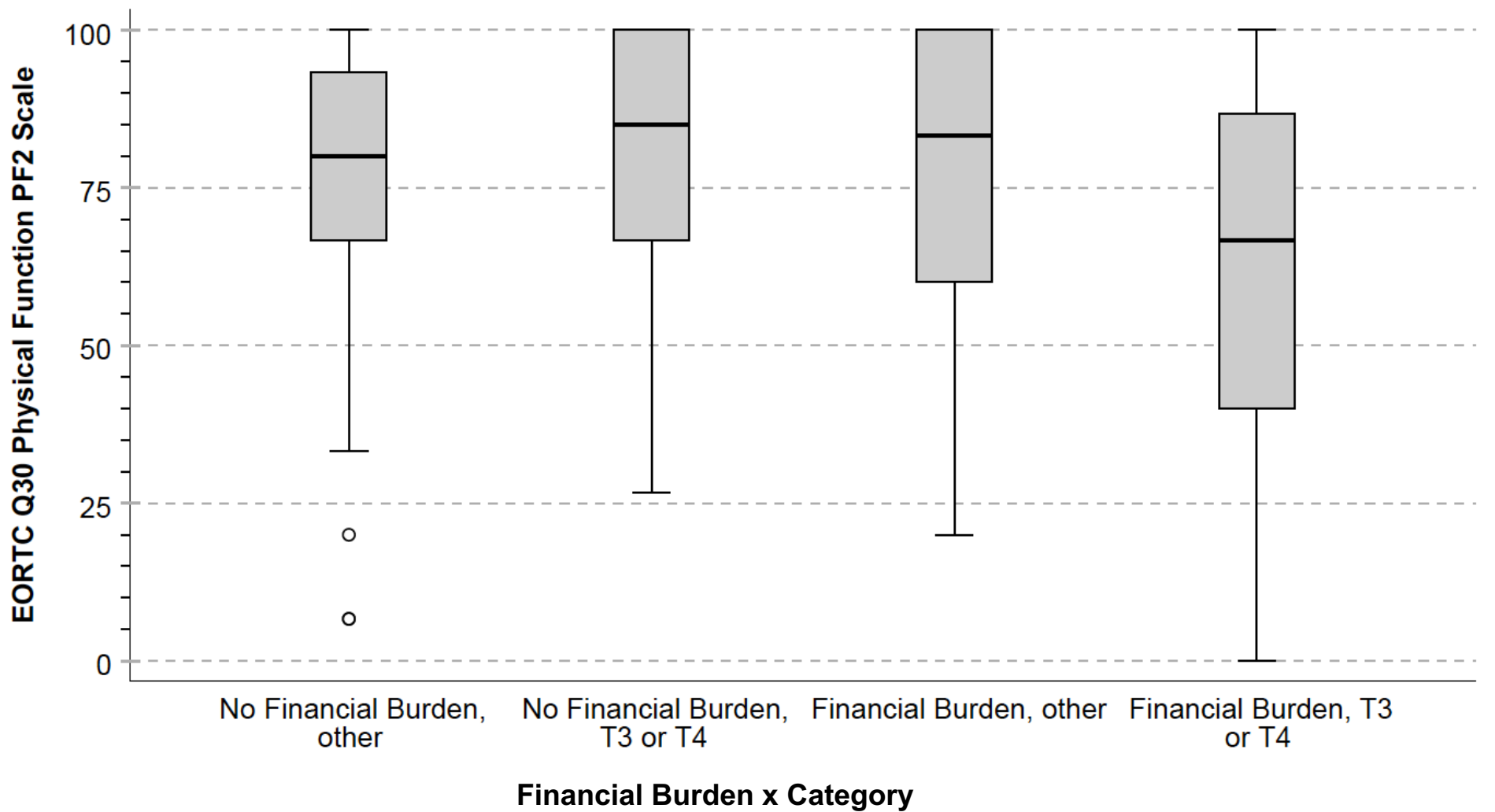

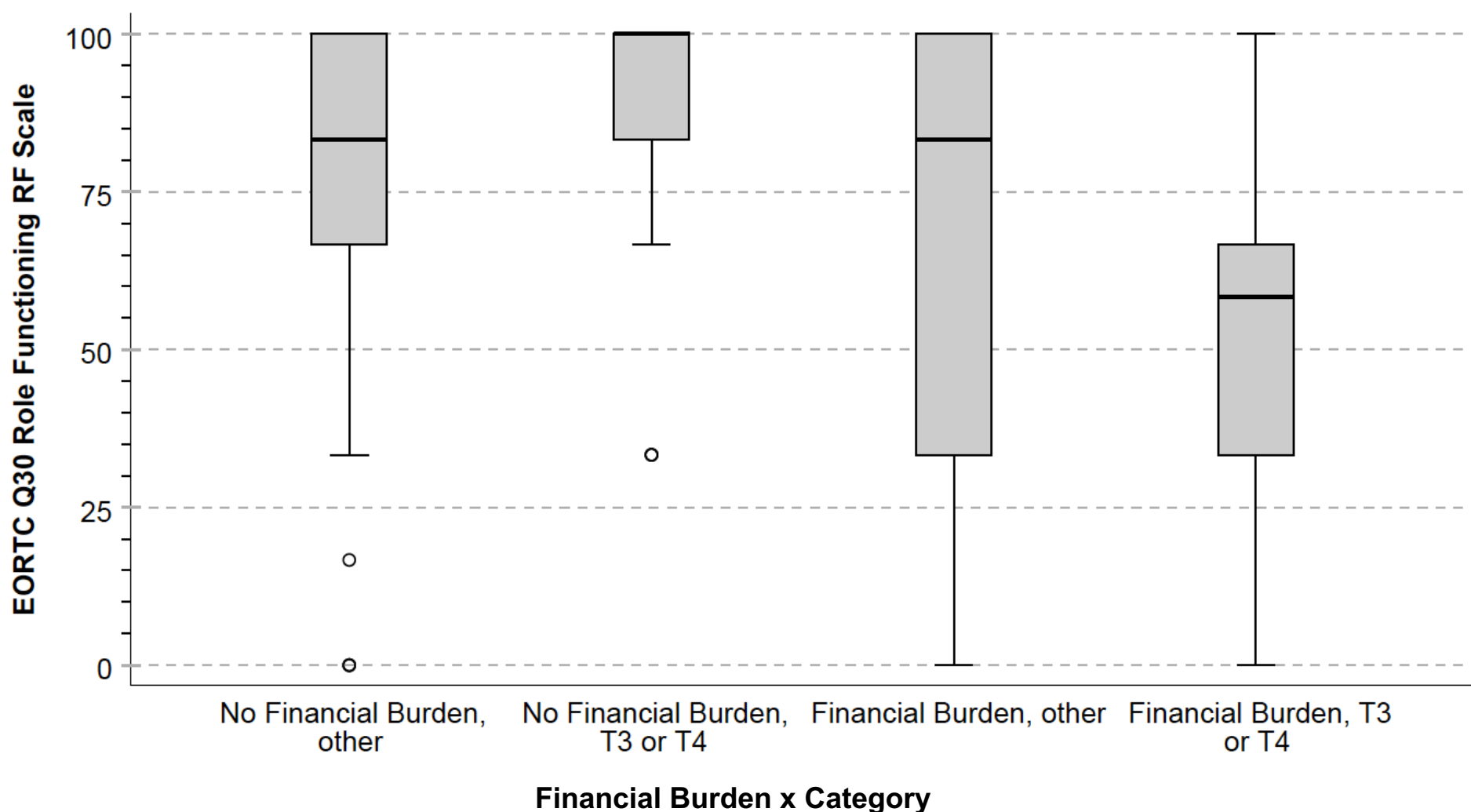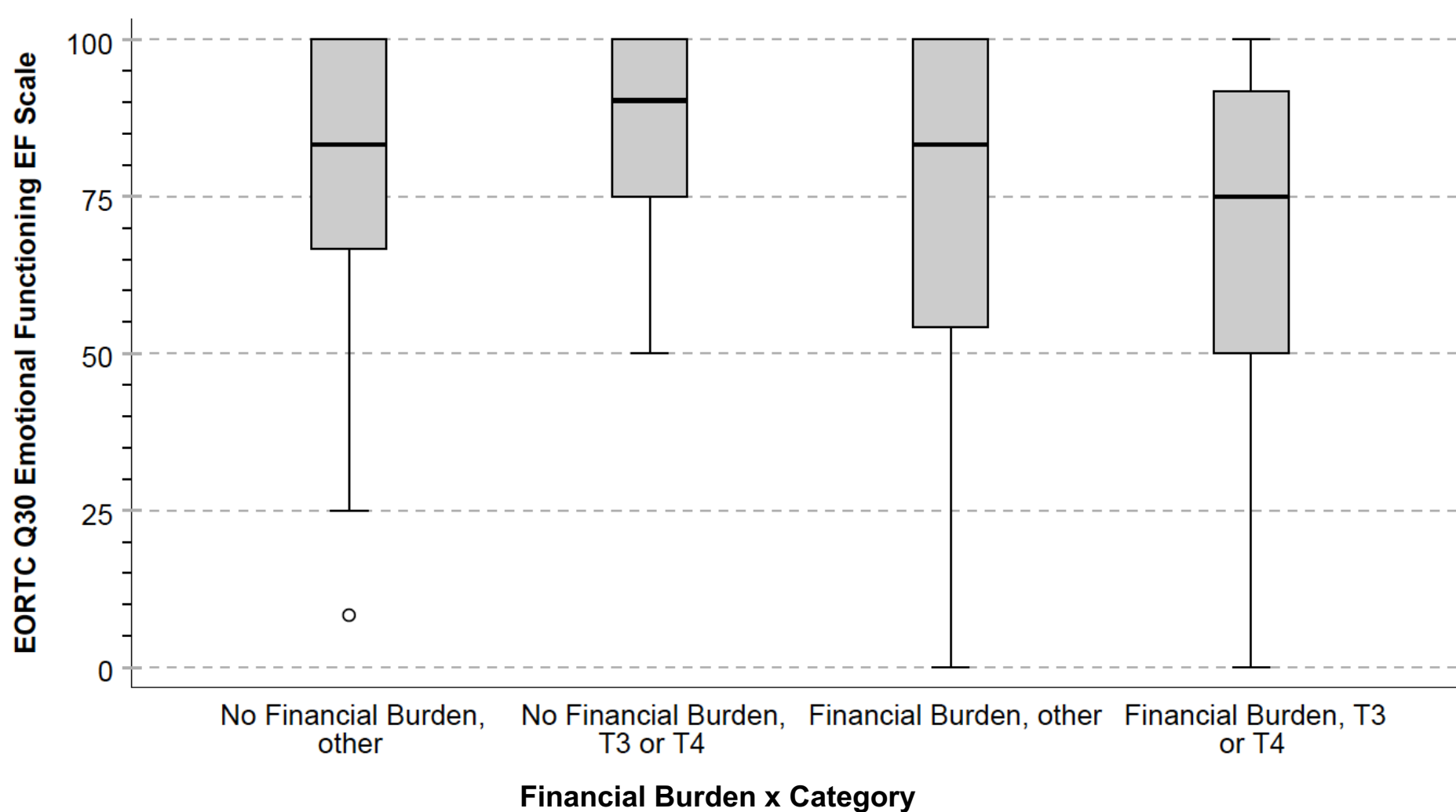

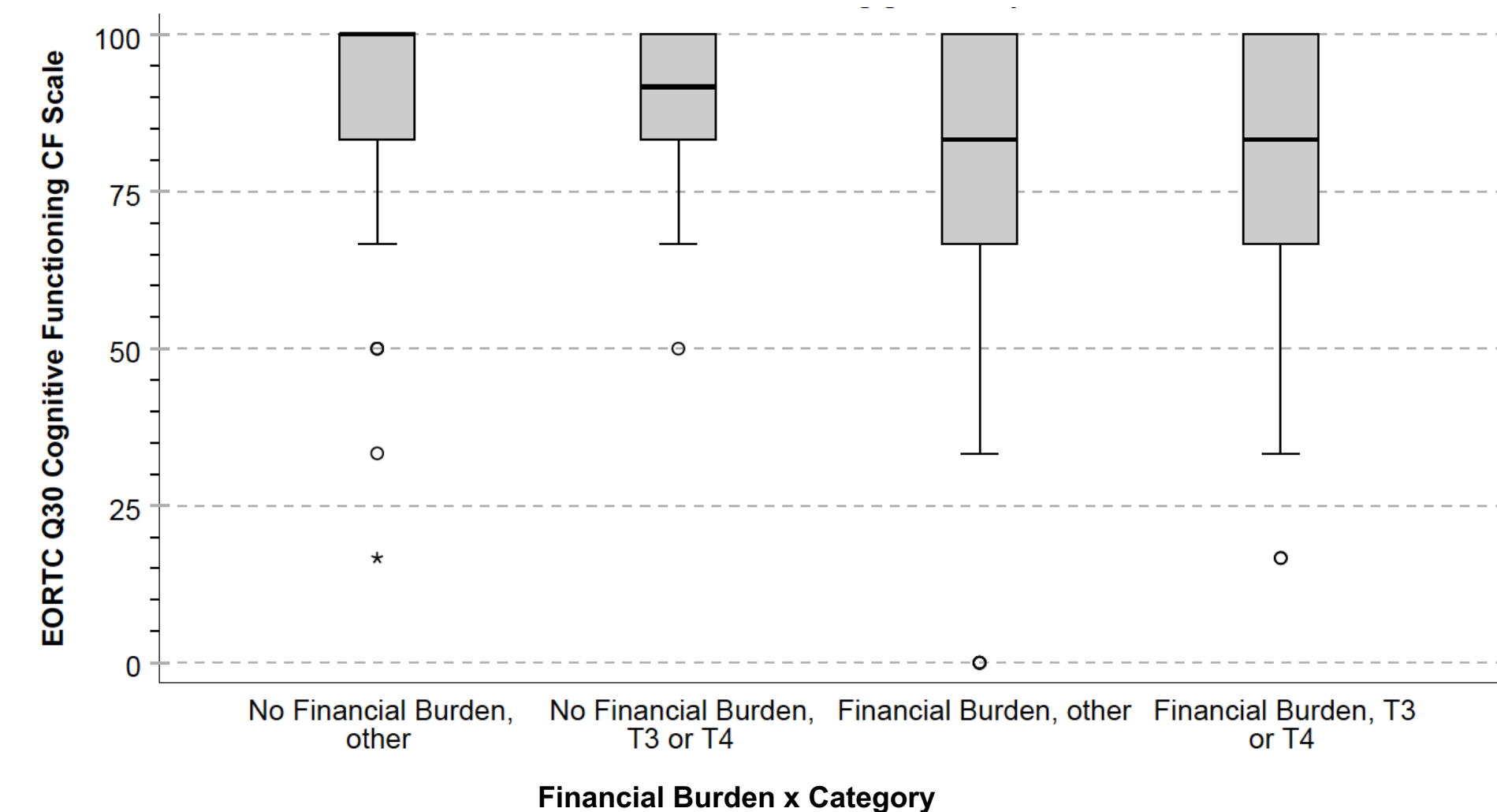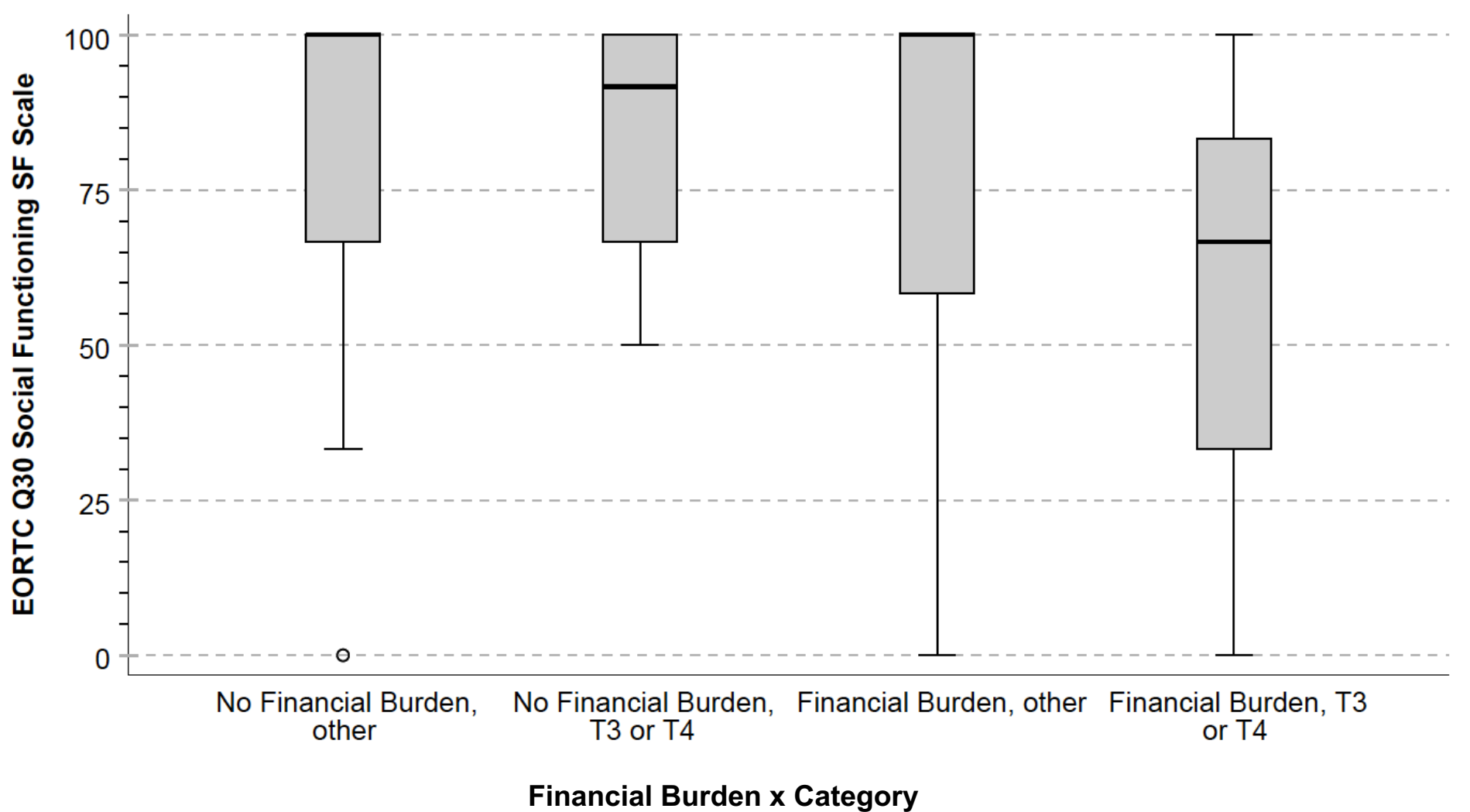

**Kruskal-Wallis test for independent samples**

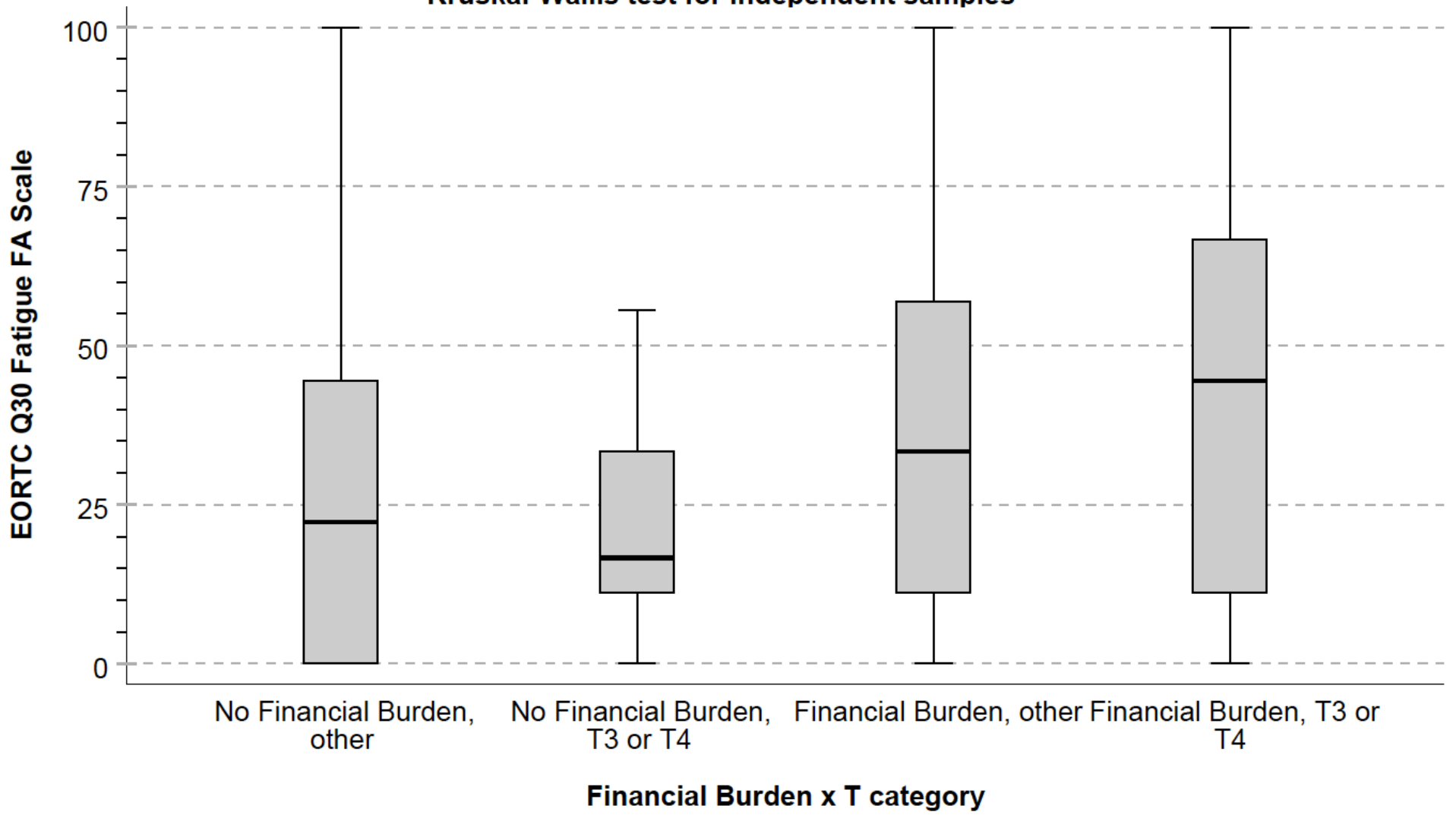

**Kruskal-Wallis test for independent samples**

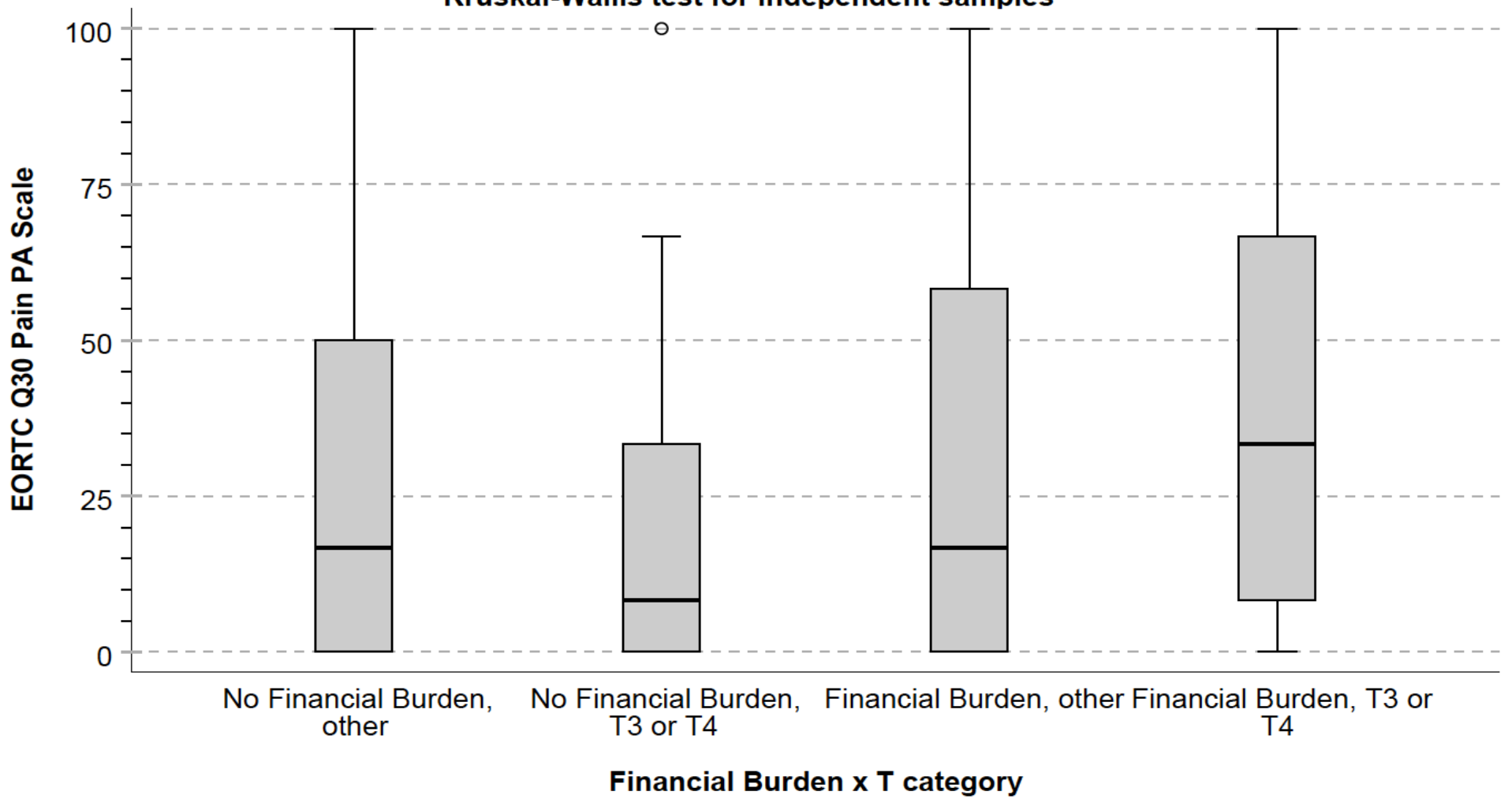

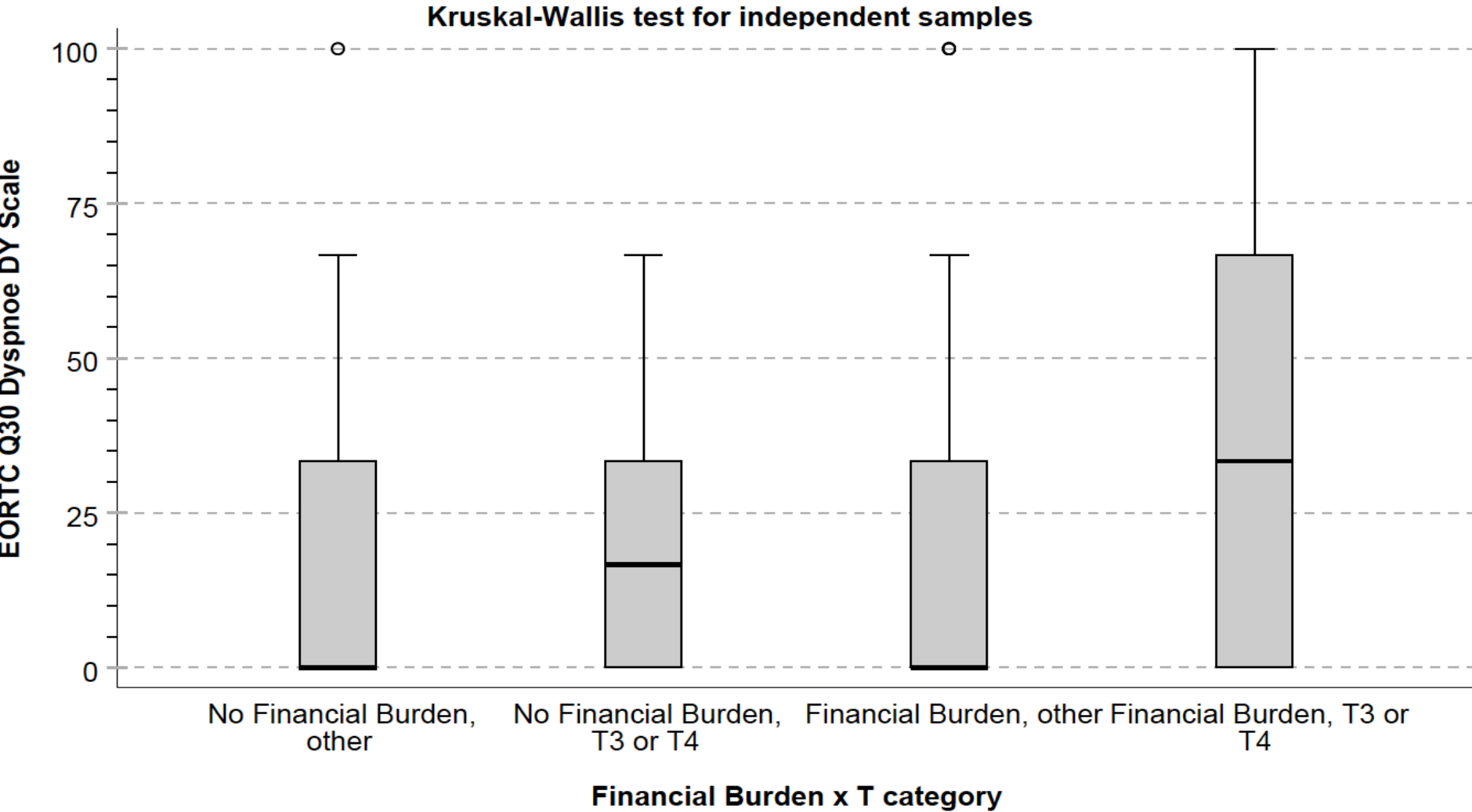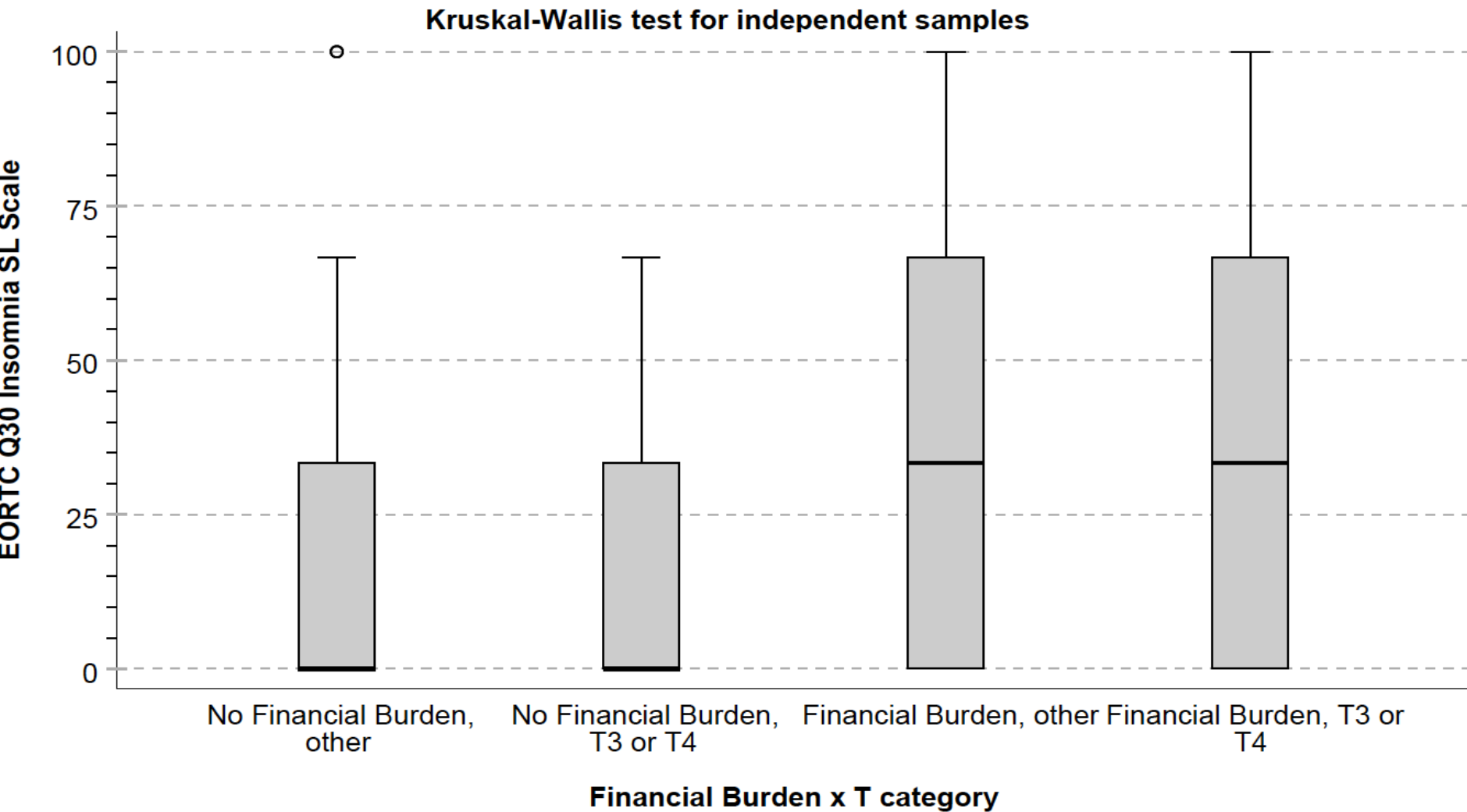

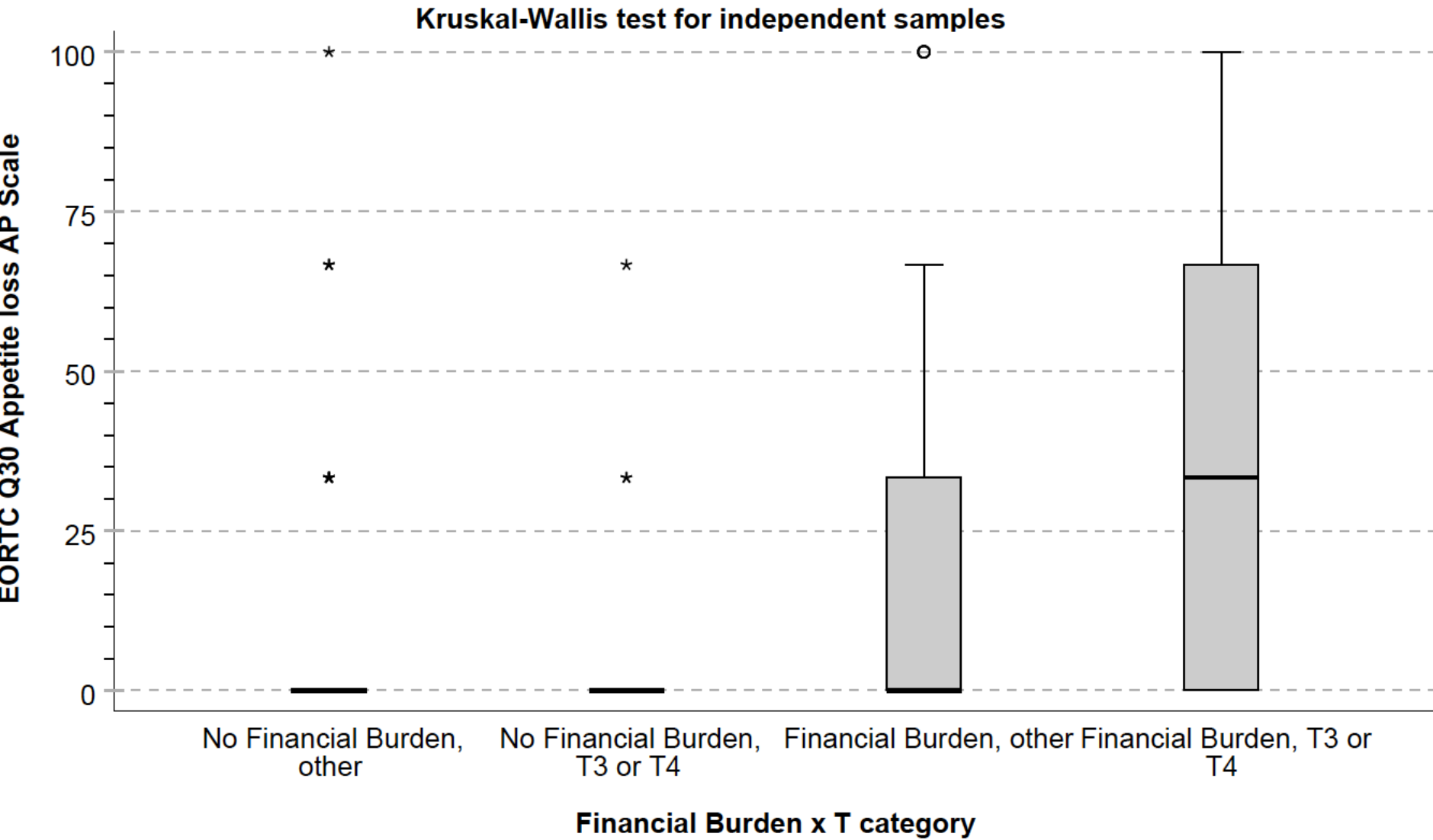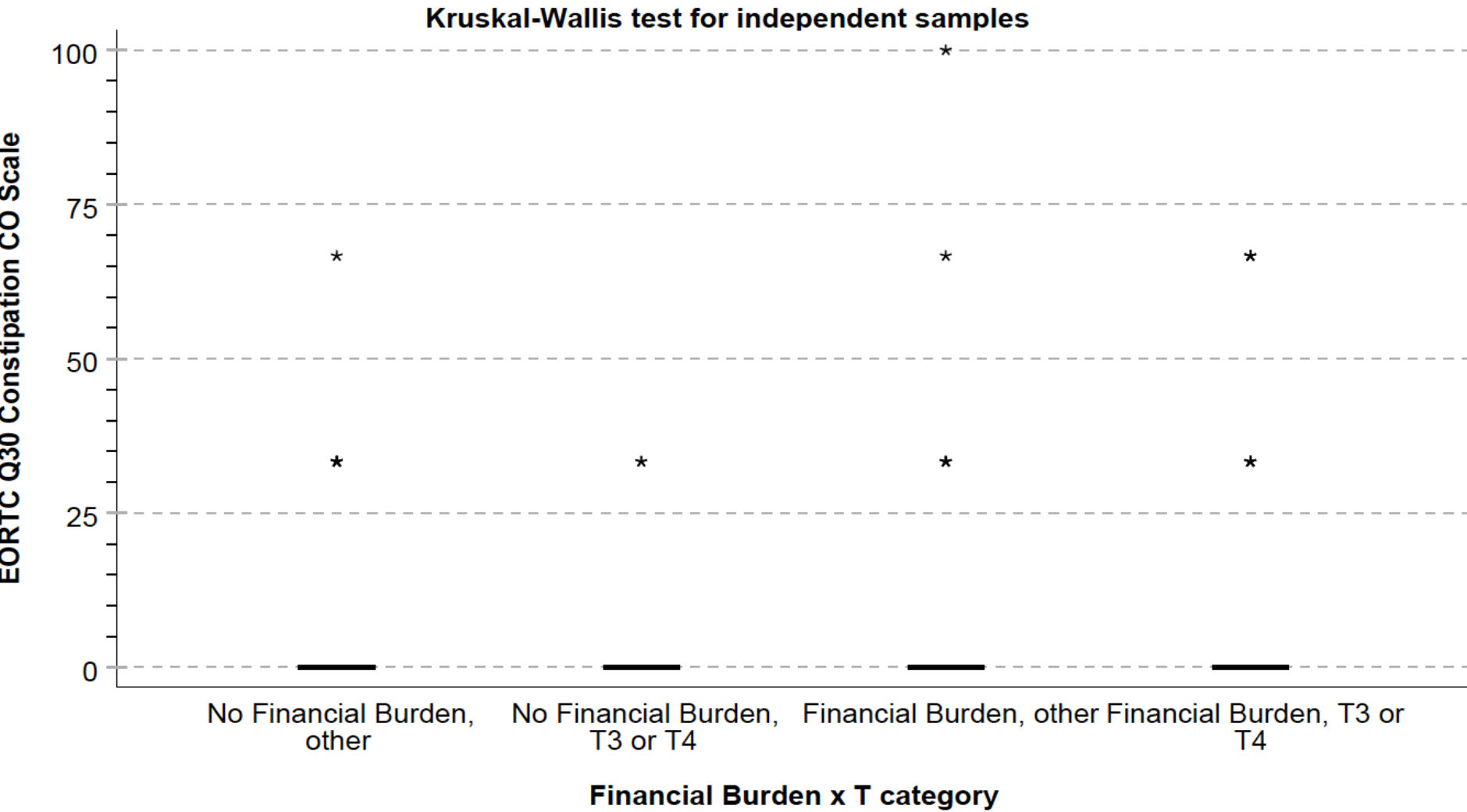

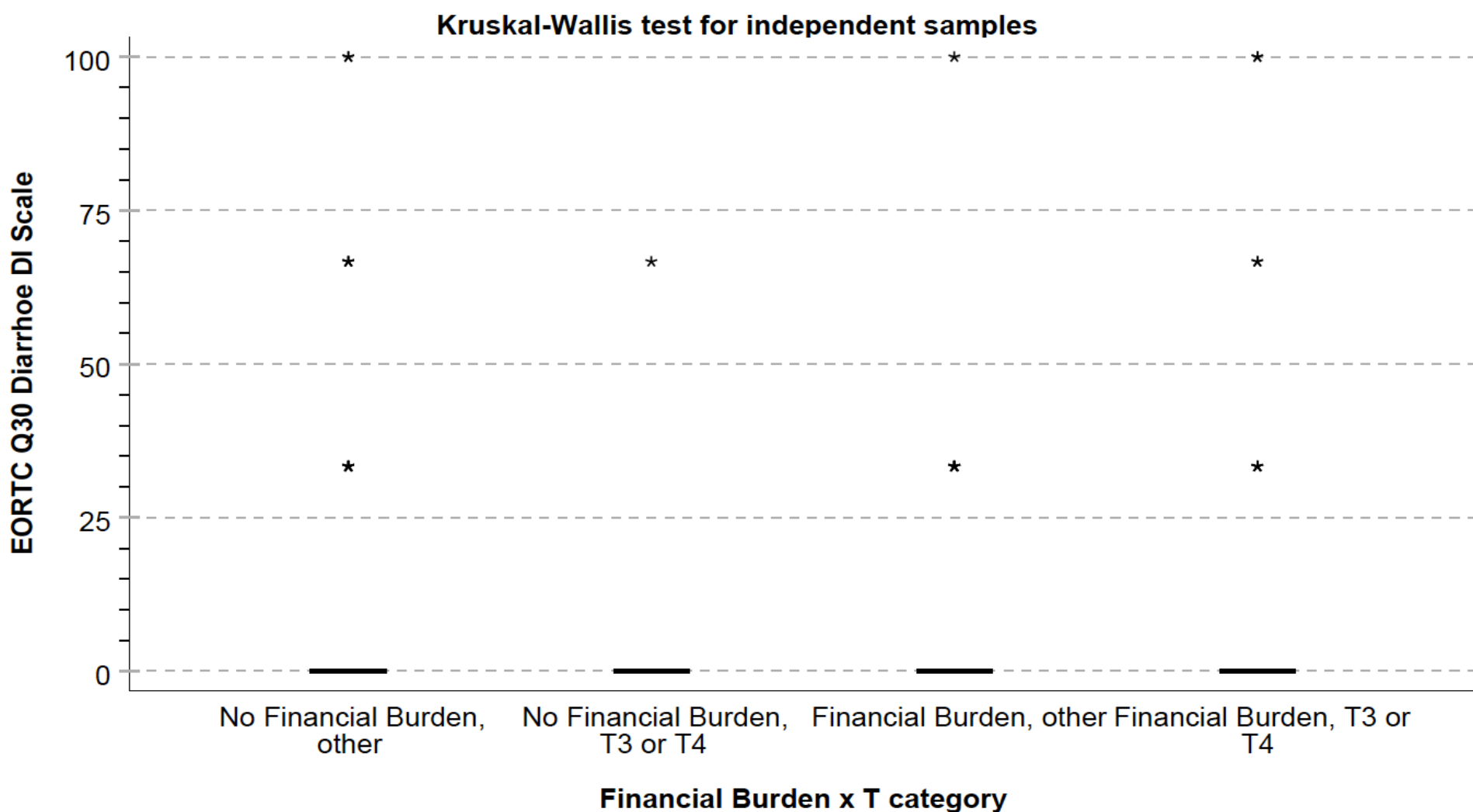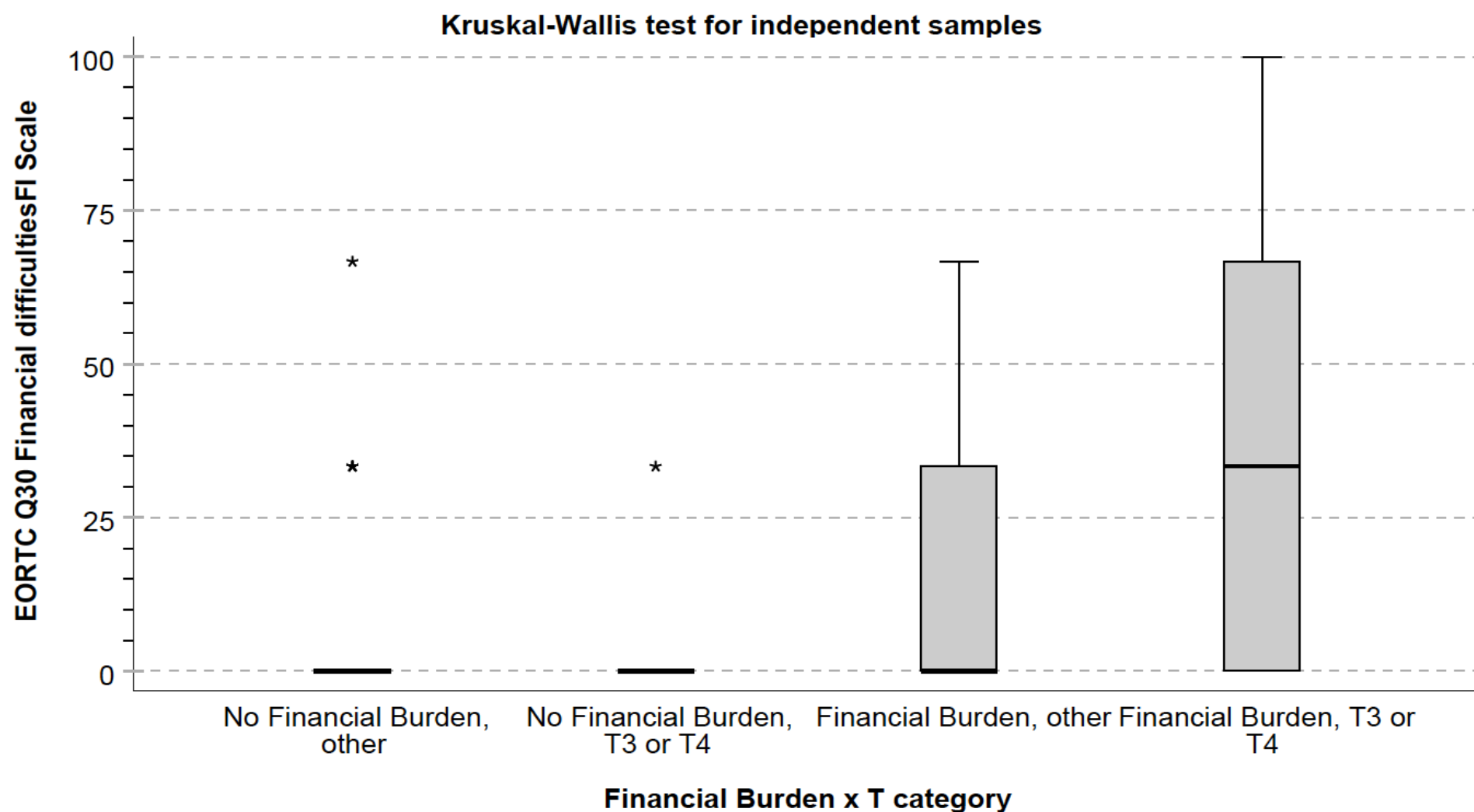

Supplement: Supplementary file 3 — Supplementary Material 3 [file 12885_2025_13927_MOESM3_ESM.pdf]
