# Supplementary material for: The impact of financial burden on quality of life among German head and neck cancer survivors
Source: BMC Cancer. 2025 Mar 20;25:514. doi: 10.1186/s12885-025-13927-1 (PMC11927114; doi:10.1186/s12885-025-13927-1)
Supplement: Supplementary file 4 — Supplementary Material 4 [file 12885_2025_13927_MOESM4_ESM.docx]

**Patient questionnaire**

**"Economic impact of head and neck tumors and their therapy"**

**A) Socio-demographic data**

1. Gender

* male

* female

2. How old are you?

____ years

3. What is your marital status?

* single

* married

* living separately

* divorced

* widowed

* with life partner

* other:

4. How many people currently live in your household?

* I live alone

* I live together with my life partner

* I live together with my spouse

* I live with another adult: __

* I am a single parent

Children living in the household: __ (number)

5. Your highest school qualification

* no degree

* Secondary school certificate/elementary school

* Secondary school

* University entrance qualification

* Other:__________

6. What vocational training do you have?

* Apprenticeship

* Vocational school (master craftsman school, technical school, vocational academy)

* Technical secondary school certificate

* University degree

* No vocational training

* Other vocational training: _


7. Do you suffer from other illnesses in addition to your cancer?

* yes, namely: ____________
* no


8. What kind of health insurance do you have?
* Private
* Statutory

* Private supplementary insurance
* different, namely:_____

**B) Professional activity and income**

9. What was your employment status at the time of the tumor diagnosis?

* employed employed

* self-employed or freelance

* civil servant

* part-time employed

* not gainfully employed

* retired

**If you were already retired at the time of the tumor diagnosis, please continue with section C- question 18**

10. What was your net income before the tumor was diagnosed?

* no own income

* under 500 Euro

* 501- 1000 Euro

* 1001 - 1500 Euro

* 1501 - 2000 Euro

* 2001 - 2500 Euro

* 2501 - 3000 Euro

* 3001 - 3500 Euro

* over 3500 Euros

11. What is your current employment status?

* employed

* self-employed or freelance

* civil servant

* part-time employed

* not gainfully employed

* retired

a) If your employment status has changed between tumor diagnosis and today, what is the reason?

* Retirement due to age

* Retirement due to tumor diagnosis

* Career change due to tumor diagnosis

* Career change for other reasons

* Job loss due to tumor diagnosis

* Job loss for other reasons

* Reduction in hours due to tumor diagnosis

* Reduction in hours for other reasons

* Permanent sick leave due to tumor diagnosis

* Permanent sick leave for other reasons

* Another reason: _______

b) If you are currently employed - is your employment contract temporary or permanent?

* I have a fixed-term employment contract

* I have a permanent employment contract

c) If you are not employed, what is the reason for this?

* I am looking for work

* I cannot be gainfully employed due to illness

* I do not wish to be gainfully employed

* Miscellaneous

12. How many months have you been on sick leave?

* _____months

* I am not on sick leave

13. Do you currently have your own income?

* yes

* no

b) If so, what is your own current monthly net income, i.e. the amount left over after deducting taxes and social security contributions?

* under 500 Euro

* 501- 1000 Euro
* 1001 - 1500 Euro
* 1501 - 2000 Euro
* 2001 - 2500 Euro
* 2501 - 3000 Euro
* 3001 - 3500 Euro
* over 3500 Euros


14. Are other people dependent on your income?
* yes, namely:___ (number) other persons
* no, just me

15. What is your current total monthly net disposable family income?

* 501- 1000 Euro

* 1001 - 1.500 Euro

* 1501 - 2000 Euro

* 2001 - 2500 Euro

* 2501 - 3000 Euro

* 3001 – 3500 Euro

* 3501- 4000 Euro

* 4001 - 4500 Euro

* 4501- 5000 Euro

* over 5000 Euro

16. Have you lost income as a result of your tumor disease?

* Yes

* no

a) If so, how much per month?

* under 100 Euro

* 100 - 200 Euro

* 201 - 500 Euro

* 501 - 800 Euro

* 801 -1200 Euro

* over 1200 Euros

b) If so, do you have the possibility of compensating for these losses?

(multiple answers possible)

* yes, via credit

* yes, via savings or assets

* yes, via other

* no

17. Did you apply for a pension after the tumor was diagnosed?
* Yes
* no

a) If so, how did this come about?
* My health insurance company has asked me to
* My employer has asked me to
* I made the request on my own initiative

**C) Expenses since tumor disease**

18. Do you have higher expenses as a result of your tumor disease? (e.g. cab fares, co-payment for medicines, treatments)

* yes

* no

a) If so, how much per month?

* under 100 Euro

* 100 - 200 Euro

* 201 - 500 Euro

* 501 - 800 Euro

* 801 - 1200 euros

* over 1200 euros

b) If so, how do you incur higher expenses?

(multiple answers possible)

* Additional payments

* Travel costs

* Utilization of domestic help

* Treatments and medication that are not covered by health insurance

* Miscellaneous

19. Has your tumor disease led to a deterioration in your overall living conditions?

* yes

* no

a) If yes, where specifically do you need to make savings? (multiple answers possible)

* in my leisure activities (e.g. going to the cinema, vacations)
* with my diet/food
* in the home/household (e.g. rent, electricity, heating)
* for medical treatments/additional services
* miscellaneous ______________

20. Which of the following benefits do you currently receive?

* Wage/salary

* Pension

* Sickness benefit

* Reduced earning capacity pension

* ALG I

* ALG II/Hartz IV

* Basic security

* Social assistance

* Benefits from occupational disability insurance

* Other, namely

21. Do you have/have you had difficulties in obtaining the above services?

receive?

* yes, because: _______

* no

22. Have you undergone rehabilitation after therapy?

* yes

* no

a) If so, how was this carried out?

* stationary

* outpatient

* Inpatient and outpatient

23. What were the most important goals of rehabilitation for you (multiple answers possible)?

* recovery from therapy / resting

* professional reintegration

* get a pension

* get to know other affected people
* physical fitness
* Other:


24. What was your relationship like at the time of rehabilitation?
* single
* married
* living separately
* divorced
* widowed
* with life partner
* Other:

We want to find out how good or bad your health is TODAY.

This scale is provided with numbers from 0 to 100.

100 is the best health you can imagine. 0 (zero) is the worst health you can imagine.

Please mark the point on the scale that best describes your health TODAY.

Now enter the number you have marked on the scale in the box below:


YOUR HEALTH TODAY =

Thank you very much for your participation!
