# Supplementary material for: The impact of financial burden on quality of life among German head and neck cancer survivors
Source: BMC Cancer. 2025 Mar 20;25:514. doi: 10.1186/s12885-025-13927-1 (PMC11927114; doi:10.1186/s12885-025-13927-1)
Supplement: Supplementary file 5 — Supplementary Material 5 [file 12885_2025_13927_MOESM5_ESM.docx]

**Supplement**

**Questionnaire 1.** The questionnaire “Economic impact of head and neck tumors and their therapy” which was used to assess financial burden (inspired by the patient survey by Mehlis et al. [37])

**Table S1.** Overview about the impact of independent predictors (*Pi*) of financial burden (FB) on quality of life (QoL) according to EORTC QLQ-C30 scales. Shown are crude and Bonferroni-corrected *p*-values from Kruskal-Wallis tests for the six orthogonal comparisons each of QLQ-C30 scales in HNC patients categorized according to binary dichotomized covariates localization (larynx/hypopharynx *versus* other), stage (UICC I or II *versus* UICC III or IV), and T category (T1/T2 *versus* T3/T4) and FB (absent *versus* present).


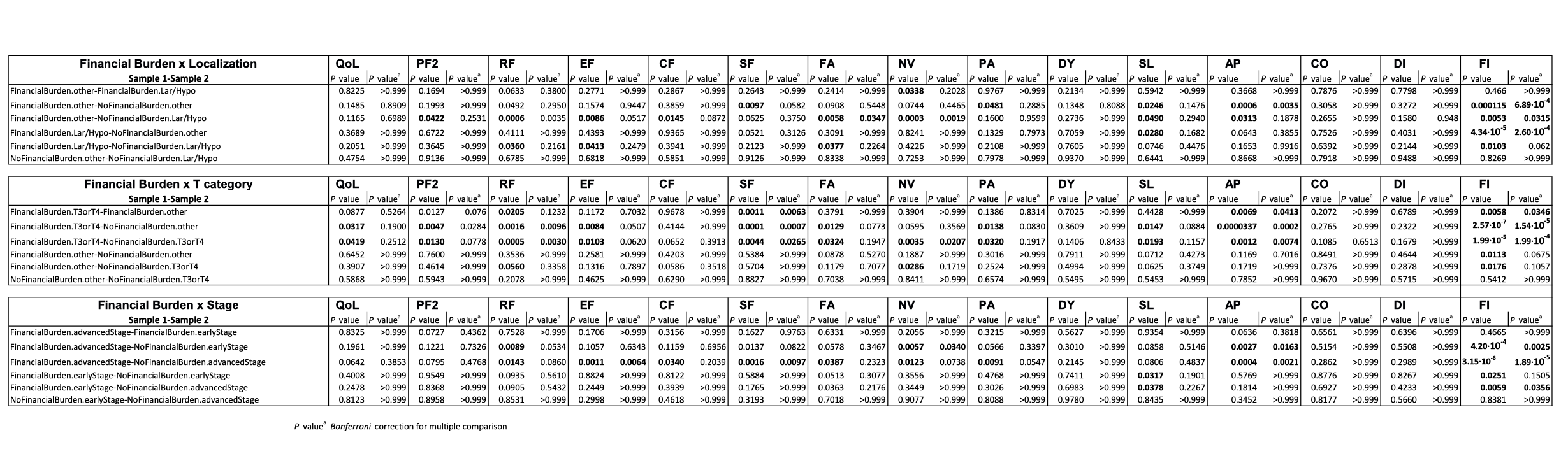


**Legends to figures**

**Figure S1.** Box plots showing the orthogonal distribution of the 15 quality of life (QoL) measures of the EORTC QLQ-C30 questionnaires in HNC patients for financial burden (absent *versus* present) and localization (larynx/hypopharynx *versus* other).

**Figure S2.** Box plots showing the orthogonal distribution of the 15 quality of life (QoL) measures of the EORTC QLQ-C30 questionnaires in HNC patients for financial burden (absent *versus* present) and stage (UICC I or II *versus* UICC III or IV).

**Figure S3.** Box plots showing the orthogonal distribution of the 15 quality of life (QoL) measures of the EORTC QLQ-C30 questionnaires in HNC patients for financial burden (absent *versus* present) and T category (T1/T2 *versus* T3/T4).
